# Disclosing a metabolic signature of cisplatin resistance in MDA-MB-231 triple-negative breast cancer cells by NMR metabolomics

**DOI:** 10.1186/s12935-023-03124-0

**Published:** 2023-12-06

**Authors:** Tatiana J. Carneiro, Ana L. M. Batista Carvalho, Martin Vojtek, Inês F. Carmo, Maria Paula M. Marques, Carmen Diniz, Ana M. Gil

**Affiliations:** 1 Department of Chemistry and CICECO –Aveiro Institute of Materials, University of Aveiro, 3810-193 Aveiro, Portugal; 2 Molecular Physical-Chemistry R&D Unit, Department of Chemistry, University of Coimbra, 3004-535 Coimbra, Portugal; 3 LAQV/REQUIMTE, Laboratory of Pharmacology, Department of Drug Sciences, Faculty of Pharmacy, University of Porto, 4150-755 Porto, Portugal; 4 Department of Life Sciences, Faculty of Science and Technology, University of Coimbra, 3000-456 Coimbra, Portugal

**Keywords:** Triple negative breast cancer, MDA-MB-231 cell line, Cisplatin resistance, Metabolic profiling, Metabolomics, Nuclear magnetic resonance

## Abstract

This work compared the metabolic profile of a parental MDA-MB-231 cisplatin-sensitive triple negative breast cancer (TNBC) cell line with that of a derived cisplatin-resistant line, to characterize inherent metabolic adaptations to resistance, as a means for marker and new TNBC therapies discovery. Supported by cytotoxic, microscopic and biochemical characterization of both lines, Nuclear Magnetic Resonance (NMR) metabolomics was employed to characterize cell polar extracts for the two cell lines, as a function of time (0, 24 and 48 h), and identify statistically relevant differences both between sensitive and resistant cells and their time course behavior. Biochemical results revealed a slight increase in activation of the NF-κB pathway and a marked decrease of the ERK signaling pathway in resistant cells. This was accompanied by lower glycolytic and glutaminolytic activities, possibly linked to glutamine being required to increase stemness capacity and, hence, higher survival to cisplatin. The TCA cycle dynamics seemed to be time-dependent, with an apparent activation at 48 h preferentially supported by anaplerotic aromatic amino acids, leucine and lysine. A distinct behavior of leucine, compared to the other branched-chain-amino-acids, suggested the importance of the recognized relationship between leucine and in mTOR-mediated autophagy to increase resistance. Suggested markers of MDA-MB-231 TNBC cisplatin-resistance included higher phosphocreatine/creatine ratios, hypotaurine/taurine–mediated antioxidant protective mechanisms, a generalized marked depletion in nucleotides/nucleosides, and a distinctive pattern of choline compounds. Although the putative hypotheses generated here require biological demonstration, they pave the way to the use of metabolites as markers of cisplatin-resistance in TNBC and as guidance to develop therapies.

**Supplementary Information:**

The online version contains supplementary material available at 10.1186/s12935-023-03124-0.

## Background

Platinum [Pt(II)]-based compounds, namely cisplatin (cDDP), carboplatin and oxaliplatin, have been extensively used in chemotherapy regimens for several types of solid tumors (*e.g.* head and neck, cervical, breast, ovarian, testicular and colon) [1, 2]. Nevertheless, the clinical use of these drugs has been limited due to their acute toxicity and associated tumor resistance [3]. Indeed, Pt(II)-resistance seriously hinders treatment efficacy, with initial response rates of *ca.* 20% and 70–90% for intrinsic and acquired resistances, respectively [4], the latter characterized by cancer recurrence within 6 months after first-line treatment (*ca.* 80% [5]). While Pt(II)-intrinsic resistance is common in colorectal and non-small lung cancers, acquired resistance is described in gynecological cancers [6], testicular, head and neck [4] and breast cancer (BC) [7]. Generally, Pt(II)-acquired resistance is a multifactorial condition believed to involve an adaptive response of tumor cells in terms of at least one of the following molecular events: (i) improvement of DNA repair mechanisms to remove DNA-drug adducts (*e.g.* nucleotide/base excision and mismatch repair) [3]; (ii) reduction of intracellular bioavailability of Pt(II)-drugs through regulation of its influx and/or efflux by selected cell membrane transport systems (*e.g.* copper transporters, Ctr1 and Ctr2, and ATP binding cassette transporters) [8]; (iii) drug inactivation or decreased drug bioavailability induced by Pt(II) coordination to sulfur-containing biomolecules (*e.g.* cysteine/methionine proteins, glutathione, or metallothionein [7, 9]); (iv) alteration in signaling pathways to promote anti-apoptotic behavior of tumors cells (*e.g.* MAPK/ERK, PI3K/AKT, NF-κB and FAS/FASL) [10]; and (v) improvement of cancer stemness progression induced by self-renewal and differentiation of cancer stem cells subpopulations [10]. Ultimately, it is believed that the effect of these resistance mechanisms is the metabolic reprogramming of resistant cells, which become more strongly reliant on glucose, glutamine and fatty acids [11]. Thus, it is important to characterize the inherent metabolic traits of Pt(II)-resistant cells, as well as their response to metallo-agents, in an attempt to develop new treatment strategies able to circumvent resistance and, therefore, lead to improved patient outcomes.

Untargeted metabolomics (either using mass spectrometry (MS) or nuclear magnetic resonance (NMR) spectroscopy) is an invaluable analytical strategy to access information on such metabolic rewiring, for instance through comparison of the metabolic profiles of Pt(II)-sensitive or Pt(II)-resistant cells. Up to this date, in vitro metabolomic studies have mainly focused on ovarian cancer [12–15] and colon cancer [16, 17]. Such studies have either only characterized the metabolism of untreated resistant cells compared to sensitive cells [12, 15, 17], or also considered the effects of exposure to cDDP [13, 14], oxaliplatin [13, 16] or carboplatin [13] on both cell lines (sensitive and resistant). The comparison of untreated resistant and sensitive cell lines is important to enable the identification of inherent mechanisms later reflected in cell behavior when under cDDP exposure. In general, untreated resistant cancer cells (namely, of colon and ovarian cancers) have been shown to exhibit a higher glycolytic activity directed towards lactic fermentation (Warburg effect) or the pentose phosphate pathway (PPP), leading to reduced oxidative phosphorylation (OXPHOS) [15–17]. In addition, more active glutaminolysis has been observed in these cases (glutamine functioning as a carbon source and maintaining redox homeostasis), as well as a less active cysteine/methionine metabolism and a reduced polyamine catabolism [12, 16, 17]. Disruptions in cell membrane metabolism have also been reported for an untreated resistant ovarian cancer cell line (namely expressed by increased levels of phosphocholine (PC) and glycerophosphocholine (GPC) and decreased levels of choline) [13], which suggest membrane changes to later mediate Pt(II) cellular uptake upon exposure. Furthermore, distinct variations in the biosynthesis of the reduced form of glutathione (GSH) and other metabolites related to the cellular antioxidant defense were observed for different untreated Pt(II)-resistant ovarian cancer cell lines, namely, increased levels of GSH and taurine in C200 cells [12] and A2780 [13] cells, respectively, while GSH, hypotaurine and taurine levels were found to be decreased in PEA2 cells [14].

Regarding breast cancer, the most prevalent cancer among the female population (affecting 2 M (24.5%) women worldwide) [18], to the best of our knowledge, no metabolomic studies have addressed Pt(II)-resistance, although targeted biochemical measurements have investigated resistance to several non-metallic drugs, such as tamoxifen (endocrine agent) [19–22] and doxorubicin (anthracycline agent) [23, 24] in BC luminal A subtype (MCF-7 cells). Furthermore, in spite of the aggressive and metastatic nature of triple-negative breast cancer (TNBC) [25], commonly treated with Pt(II)-based therapies, the few existing metabolomic reports have, to our knowledge, only addressed sensitive cell lines, to describe the impact of cDDP (namely on MDA-MB-231, MDA-MB-468 or SUM-159PT cell lines), compared to other agents such as tamoxifen, doxorubicin and a Cu(II)-chelate [26–28]. Metabolomics has also addressed the metabolic response of MDA-MB-231 cells to cDDP alone and in combination with valproic acid, an antiepileptic drug with anticancer properties as histone deacetylase inhibitor, believed to influence resistance acquired by epigenetic modifications [29].

This paper presents, for the first time to the authors’ knowledge, an untargeted metabolomics study of the polar metabolome of untreated MDA-MB-231 parental (cDDP-sensitive) cells and derived MDA-MB-231/R cells (cDDP-resistant), the latter having been previously established as a model of acquired cDDP-resistance [30]. An NMR metabolomics strategy was applied, with a view to assess the short-term (up to 48 h) metabolic dynamics of each untreated cell line, aiming at identifying metabolic traits characterizing cDDP-acquired resistance in TNBC. The unveiled metabolic characteristics of cDDP-resistance contribute to the identification of the mechanisms involved in the process, thus paving the way for early prediction of cDDP response and aid the development of new strategies to overcome cDDP-resistance.

## Methods

### Cell culture

The human TNBC cell line MDA-MB-231 (ATCC HTB-26; absence of estrogen and progesterone receptors, HER2 overexpression) was purchased from ATCC (Manassas, VA, USA). BC cells were cultured in DMEM-HG cell growth medium supplemented with 10% (v/v) FBS and maintained under a humidified atmosphere of 5% CO_2_ at 37 °C. The cDDP-resistant cell line was established as previously described [30]. Briefly, MDA-MB-231 cells were continuously treated with increasing concentrations of cDDP (up to a maximum of 2 µM) during 6 months. When a consistent cell growth rate was achieved, in the presence of cDDP, the resulting cell line, designated as MDA-MB-231/R, was stored at − 80 °C. All subsequent experiments were performed within 10 passages, for both cDDP-sensitive and -resistant cell lines, maintaining the MDA-MB-231/R cell line in growth medium in the absence of cDDP. Under these conditions, the population doubling times were 25.5 ± 0.9 h and 30.6 ± 1.1 h for MDA-MB-231 (sensitive, designated as S) and MDA-MB-231/R (resistant, designated as R) cells, respectively. The two cell lines will be designated, when possible, as R and S for MDA-MB-231/R and MDA-MB-231, respectively. The cell cultures were routinely screened for mycoplasma contamination, all assays having yielded negative results.

### Cell growth assays

Cells were seeded in 96-well microplates at the cell density 1.5 × 10^4^ cells/cm^2^ (final volume 200  µL/well) and left to attach for 24 h. Label-free kinetic live monitoring of cell growth was performed using LionheartFX automated microscope (BioTek, Winooski, VT, USA) with direct image acquisition of cells in microplates at 48 h. The 4X images acquired were processed using the Gen 5 Image Analysis software (BioTek, Winooski, VT, USA) that allows for identification and counting of individual cells per image. IC_50_ (half maximal inhibitory concentration) values were calculated for both S and R cell lines incubated for 48 h with increasing concentrations of cDDP (5, 10 and 20 μM) [30].

### ERK1/2 phosphorylation assays and NF-κB phosphorylation assays

Changes in phosphorylated ERK1/2 (p-ERK) were detected using the AlphaScreen SureFire p-ERK1/2 Kit following the methods described in detail elsewhere [31, 32]. Briefly, 10 μL of total protein lysate were transferred into 384-well ProxiPlates and a mixture of acceptor beads and donor beads were added, following the manufacturer’s instructions. In addition, changes in phosphorylated NF-κB (p-NF-κB) were measured using the AlphaScreen SureFire p-NF-κB Kit, following the same protocol described above.

### Cell assays statistical analysis

In relation to cell assays, the data was expressed as the mean ± standard error of the mean (SEM), with each of three independent experiments, in triplicate (*n* = 3). Statistical analysis of cell assay results was performed using (i) non-linear regression analysis of the corresponding dose–response curves to calculate the IC_50_ values; (ii) the two-tailed Student’s t-test to compare R *vs.* S cells; (iii) one-way ANOVA to compare each cell line with the respective controls, followed by the Dunnett’s *t*-test. The GraphPad Prism 7 Software (San Diego, CA, USA) was used. A *p*-value lower than 0.05 was considered statistically significant.

### Cells collection and extraction

MDA-MB-231 parental (cDDP-sensitive) and resistant cells were seeded at a density of 3 × 10^4^ cells/cm^2^ onto 150 mm Petri dishes (ø 135.5 mm), cultured in a humidified atmosphere of 5% CO_2_ at 37 °C and allowed to adhere for 24 h. The cells were then incubated and collected at 0, 24 and 48 h, with basis on the population doubling times mentioned above. At each time-point, cells were harvested using a 0.25% (v/v) trypsin–EDTA solution, washed twice with PBS and centrifuged (300 g, 5 min, 20 °C). The cell pellets were stored at − 80 °C until analysis. Three independent experiments with triplicates were performed for each cell line and time-point.

The cellular polar extracts were obtained using a biphasic extraction method of methanol/chloroform/water previously reported [33]. Briefly, cell pellets were suspended in 650 µL of 80% (v/v) methanol-miliQ water solution, transferred to microcentrifuge tubes with 150 mg of glass beads (ø = 0.5 mm) previously weighted, and vortexed for 5 min to aid cells disruption. Subsequently, 260 µL of 100% chloroform and 260 µL of 100% chloroform plus 220 µL MiliQ water were added to samples, which were vortexed for 5 min between solvents addition. Samples were stored at −20 °C for 10 min and centrifuged (2,000 g, 15 min, room temperature). The aqueous phase was collected into a new tube, vacuum-dried and stored at − 80 °C until further analysis. All samples and reagents were kept in ice during the extraction procedure. Before NMR analysis, the dry aqueous extracts were suspended in 650 µL of 100 mM sodium phosphate buffer (pH 7.4, in D_2_O containing 0.25% 3-(trimethylsilyl)-propionic-2,2,3,3-d4 acid (TSP) for chemical shift referencing) and transferred into 5 mm NMR tubes.

### NMR spectroscopy and statistical analysis of spectra

The NMR spectra were recorded on a Bruker AVANCE III spectrometer, equipped with a 5 mm TXI probe, operating at 500.13 MHz for ^1^H observation, and at 298 K. Standard 1D ^1^H NMR spectra of aqueous extracts were acquired using a water presaturation pulse sequence (“noesypr1d” from Bruker library, Rheinstetten, Germany), with 7002.801 Hz spectral width, 32 k data points, 2.34 s acquisition time and 2 s relaxation delay and 512 scans. Prior to Fourier transformation, each free-induction decay was zero-filled to 64 k points and multiplied by a 0.3 Hz exponential line-broadening function. Spectra were manually pre-processed including phase correction, baseline adjustment and internal calibration of chemical shifts to TSP. For peak assignment, 2D NMR homonuclear total correlation (TOCSY) and heteronuclear single-quantum correlation (HSQC) spectra were acquired for selected samples, along with comparison with existing literature and spectral databases, such as Bruker BIOREFCODE (AMIX-viewer 3.9.14, Bruker Biospin, Rheinstetten, Germany), human metabolome database (HMDB) [34] and Chenomx NMR Suite (Chenomx Inc, Edmonton, AB, Canada).

The unidimensional proton NMR spectra were converted into matrices (AMIX 3.9.14, Bruker Biospin, Rheinstetten, Germany), excluding methanol (δ 3.36, singlet) and water (δ 4.4–5.4) spectral regions. The spectra were aligned by recursive segment-wise peak alignment (RSPA) to minimize chemical shift variations (Matlab 8.3.0, The MathWorks Inc., Natick, Massachusetts, USA), and normalized to total spectral area to account for different cells numbers. Multivariate analysis was carried out using unsupervised principal component analysis (PCA) and supervised partial least squares–discriminant analysis (PLS-DA), upon unit variance (UV) scaling (SIMCA-P 11.5; Umetrics, Umeå, Sweden). PLS-DA models with corresponding values of predictive power (Q^2^) higher than 0.50 were considered statistically robust. PLS-DA loadings were back-transformed, multiplying each variable by its standard deviation, and colored according to variable importance to the projection (VIP) (Matlab 8.3.0, The MathWorks Inc., Natick, MA, USA). The respective loading plots revealed the resonances relevant for class separation, which were selected for area integration (AMIX 3.9.14, Bruker BioSpin, Rheinstetten, Germany), normalization, and variation assessment by univariate analysis. Univariate analysis of metabolites combined effect size (ES) [35] and statistical significance (Shapiro–Wilk test to assess data normality, Student’s *t*-test or Wilcoxon test for normally distributed or non-normally distributed data, respectively) (R statistical software). For multiple testing, *p*-values of significantly changed metabolite levels (|ES|> ES error and *p* < 0.05) were corrected by false discovery rate (FDR), based on the Benjamini and Hochberg method [36]. Significant metabolite differences between S and R cell lines were all confirmed by posterior visual inspection of the spectra, and putatively interpreted based on information derived from the Kyoto Encyclopedia of Genes and Genomes (KEGG) database [37].

## Results

### Cytotoxic, microscopic and biochemical characterization of MDA-MB-231/R (R) compared to MDA-MB-231 (S) cells

**Fig. 1:**
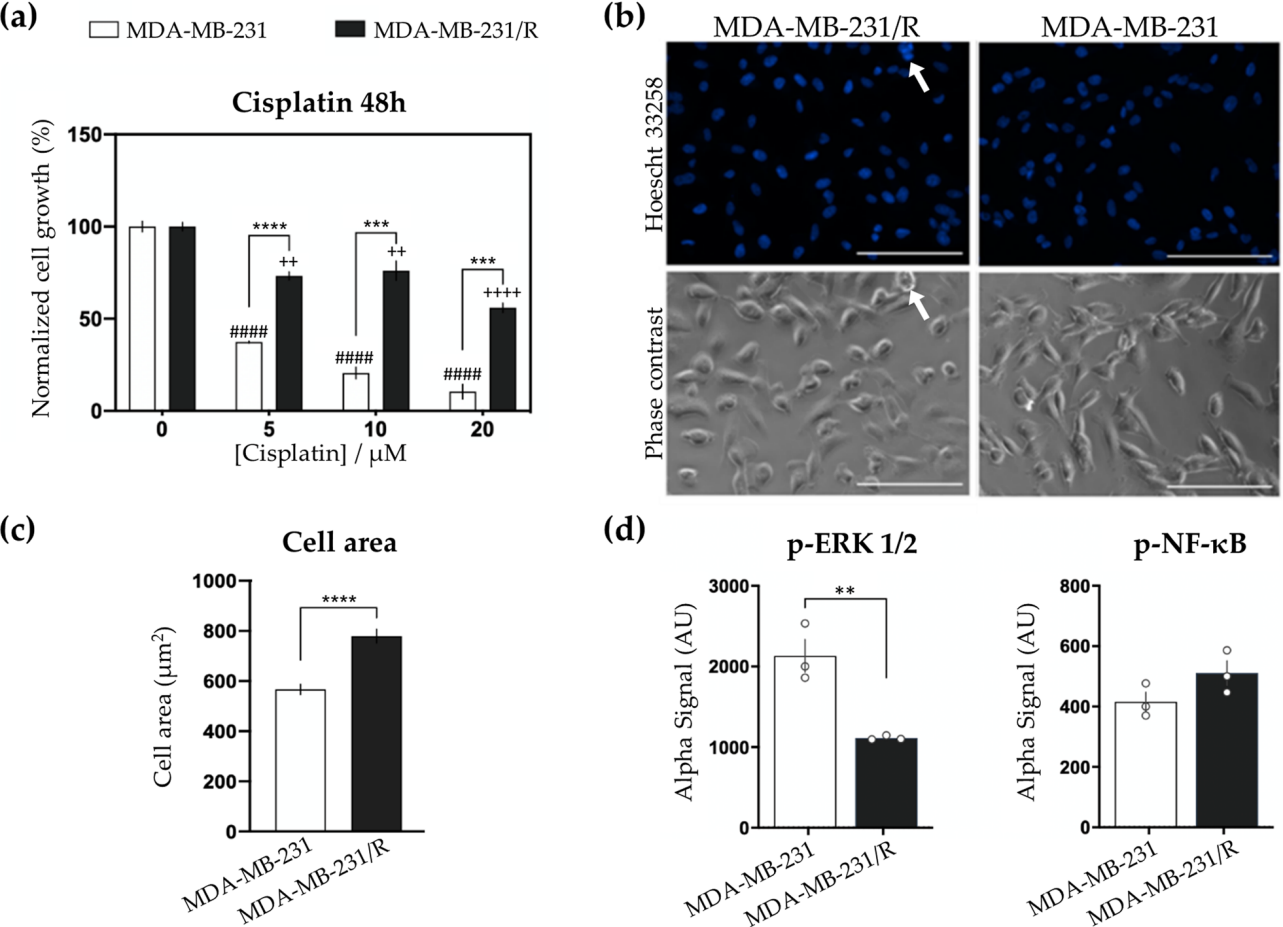
Cytotoxicity, microscopy and biochemical comparison of MDA-MB-231 and MDA-MB-231/R cells. **a** Proliferation of MDA-MB-231 and MDA-MB-231/R cells treated with 0.5, 10 and 20 μM of cisplatin for 48 h. Values are expressed as mean ± SEM, *n* = 3 experiments (triplicates). Significant differences from control (MDA-MB-231): *p* < 0.0001 (####); Significant differences from control (MDA-MB-231/R): *p* < 0.01(+ +); *p* < 0.0001 (+ +  + +); Significant differences from MDA-MB-231 cells: *p* < 0.001 (***), *p* < 0.0001 (****); **b** Representative photomicrographs of MDA-MB-231/R cells (left) and MDA-MB-231 (right). 4 independent experiments were carried out under a fluorescent objective lens (upper panel) or a phase-contrast objective lens (lower panel) of the LionheartFX microscope. Scale bar = 100 μm; **c** Area of polyploid giant cancer cells in MDA-MB-231 (white) and MDA-MB-231/R cells (black). Values are expressed as mean ± SEM, *n* = 3 experiments (triplicates). Significant differences from MDA-MB-231 cells: p < 0.0001 (****); **d** Phosphorylation of ERK1/2 and of NF-κB in MDA-MB-231 (white) and MDA-MB-231/R cells (black) detected using AlphaScreen Sure Fire technology. Values are expressed as mean ± SEM, *n* = 3 experiments (triplicates). Data are expressed as mean ± SEM, *n* = 3. Significant differences from MDA-MB-231 cells: p < 0.01 (**)

In order to demonstrate cDDP-resistance in R cells, the impact of increasing concentrations of cDDP (5, 10 and 20 μM) on S or R cells, incubated for 48 h, was evaluated through cell growth measurements (Fig. 1a). The results showed that S cells were highly sensitive to cDDP treatment, while the response of R cells to cDDP was considerably attenuated (Fig. 1a), with similar doubling times for both cell lines (25.5 ± 0.9 h and 30.6 ± 1.1 h for S and R cell lines, respectively). This was consistent with the reported cDDP half maximal inhibitory concentrations IC_50_ (48 h) of 1.0 μM and 32.4 μM for S and R cells, respectively [30]. The above results are consistent with data previously reported for other MDA-MB-231 cDDP-resistant human cell lines [38–40].

Morphological analysis revealed a normal spindle-shaped phenotype for both S and R cells, although R cells were characterized by an enriched portion (Fig. 1b) and a larger cell size (566.4 ± 20.7 μm^2^ in S vs. 779.4 ± 27.4 μm^2^ in R cells, p < 0.0001, Fig. 1c) of polyploid giant (cancer) cells (PGCCs) as compared to S cells. Furthermore, the basal phosphorylation profiles of ERK1/2 and NF-κB proteins in S and R cells were measured and compared (Fig. 1d), as the MAPK/ERK pathway plays a crucial role in the survival and development of tumor cells [41, 42], whereas the NF-κB pathway has also been linked to cancer survival [43]. For R cells, the basal phosphorylation of NF-κB showed an increasing tendency of nearly 17% (p = 0.1380), while a significant decrease was noted in the basal levels of phosphorylated ERK1/2 (near 50% decrease, p = 0.0077), compared to S cells (Fig. 1d).

### Overall metabolic profiling of MDA-MB-231/R (R) compared to MDA-MB-231 (S) cells

**Fig. 2:**
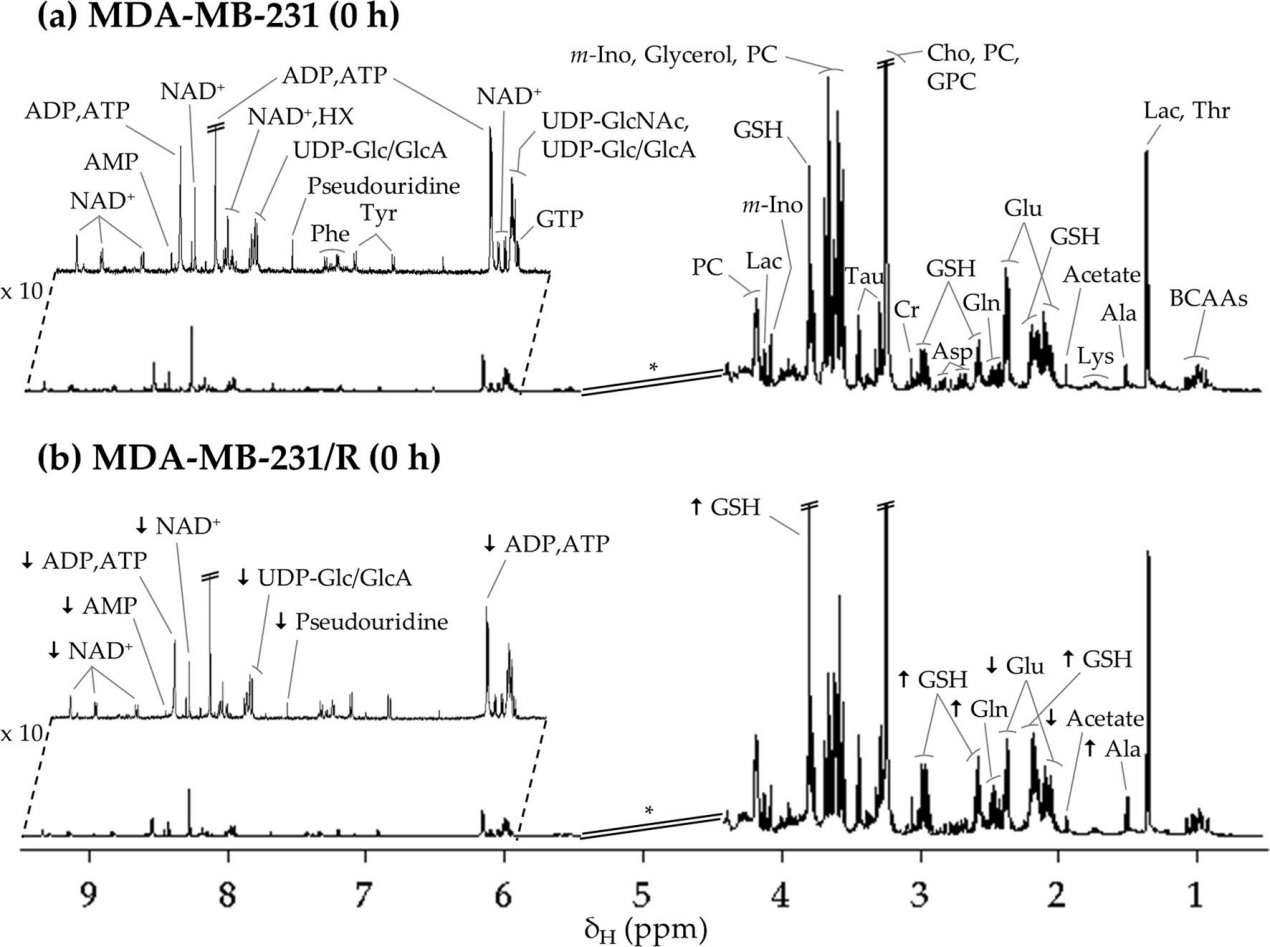
Average 500 MHz ^1^H NMR spectra of aqueous extracts from triple-negative breast cancer cells. **a** sensitive (S), MDA-MB-231, and **b** resistant (R), MDA-MB-231/R to cDDP, at the beginning of the experiment (t = 0 h). * Cut-off of water suppression region (δ 4.4–5.4), not considered in the multivariate analysis. The arrows identify metabolic variations found with visual inspection of spectra of R cells in relation to S cells. 3-letter code for amino acids; *ADP* adenosine diphosphate, *AMP* adenosine monophosphate, *ATP* adenosine triphosphate, *BCAAs* branched-chain amino acids (Ile, Leu and Val), *Cho* choline, *Cr* creatine, *GPC* glycerophosphocholine, *GSH* glutathione (reduced), *GTP* guanosine triphosphate, *HX* hypoxanthine, *m-Ino myo*-Inositol, *Lac* lactate, *NAD*^+^ nicotinamide adenine dinucleotide (oxidized), *PC* phosphocholine, *Pseudourd.* Pseudouridine, *Tau* taurine, *UDP-Glc/GlcA* uridine diphosphate-glucose/glucuronate, *UDP-GlcNAc* uridine diphosphate *N*-acetyl-glucosamine

The average ^1^H NMR spectrum of polar extracts of untreated S cells (0 h) (Fig. 2a) reflects the presence of over 20 amino acids and derivatives, 3 choline compounds (choline, phosphocholine (PC), glycerophosphocholine (GPC)), 17 nucleotides and derivatives, 8 organic acids and 4 other compounds (dimethylamine (DMA), glycerol, *m-*inositol and trimethylamine *N-*oxide (TMAO)) (see table of assignments, Additional file 1). This is consistent with previously reported NMR data for polar extracts of this cell line [27, 44]. At 0 h, some changes are apparent through visual inspection of the average spectrum of R cells (arrows in Fig. 2b), compared to that of S cells, namely increased alanine, glutathione (reduced form, GSH) and glutamine, and decreased acetate, glutamate, adenosine-mono/di/triphosphate (AMP, ADP, ATP), pseudouridine, uridine diphosphate glucose/glucuronic acid (UDP-Glc/GlcA) and nicotinamide adenine dinucleotide (NAD^+^), all of these variations having been confirmed upon statistical analysis, as described below.

**Fig. 3:**
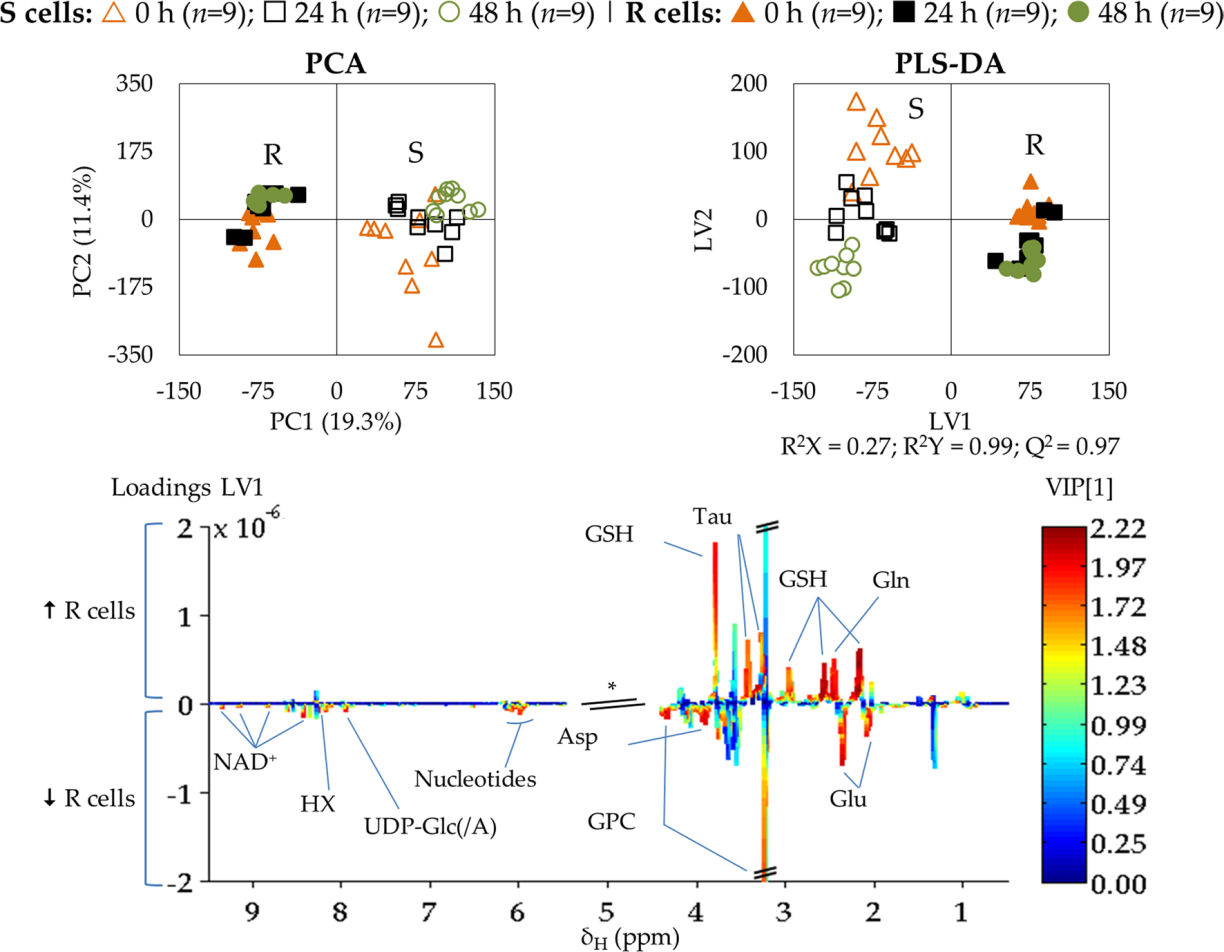
Multivariate analysis of spectra of aqueous extracts from MDA-MB-231 cells (S) *vs.* MDA-MB-231/R cells (R). Score scatter plots of PCA and PLS-DA models (top) and LV1 loadings (bottom), considering the trajectory of the three experimental time-points: t = 0 h (orange triangles, *n* = 9/cell type), t = 24 h (black squares, *n* = 9/cell type), t = 48 h (green circles, *n* = 9/cell type). Validation parameters (R^2^ and Q.^2^) are shown for the PLS-DA model. Loadings’ peak assignments are indicated for the metabolites most relevant for group separation according to the color representation of variable importance to projection color (VIP). Abbreviations as defined in Fig. 2

PCA and PLS-DA models were used to compare all R and S samples (full and open symbols, respectively, in Fig. 3, top) showing that the metabolic profile is significantly distinct between the two cell lines, independently of culture time and with slightly more dispersion for sensitive cells. PLS-DA LV1 loadings (Fig. 3, bottom) seem to indicate that some of the most consistent differences affect glutamate, glutamine, GSH, GPC, NAD^+^, taurine, aspartate, several nucleotides, UDP-Glc/GlcA and hypoxanthine (HX).

Such differences were confirmed by spectral integration and statistical assessment, which produced a more complete list of reliable differences between all R and S cells, independently of time point (Table 1). Overall, the results evidence that cDDP-resistant cells differ from sensitive cells in the levels of 38 identified compounds: 14 amino acids and derivatives, choline and GPC, 15 nucleotides and derivatives, 5 organic acids, DMA and *m*-inositol. The most significant differences (*p* ≤ 10^–10^ and |ES|≥ 2.0) in resistant cells include (i) higher levels of GSH, glutamine, phosphocreatine (PCr), taurine and lower levels of glutamate; and (ii) overall lower levels of adenine, adenosine, NAD^+^ and UDP-Glc/GlcA (Table 1, bold and underlined metabolites), although all listed variations are important (*e.g.* depletion in choline, GPC, and in a wide range of nucleosides/nucleotides), all organic acids detected (including lactate) and *m-*inositol, as they remain statistically meaningful after FDR correction.

**Table 1:** Statistically significant (|ES|> ES Error and *p* < 0.05) metabolite variations observed in the polar metabolome of MDA-MB-231/R (R) compared to MDA-MB-231 (S) (not considering time-course evolution)

Metabolite		δ_H_ (multiplicity)	R *vs.* S		
			ES ± Error		*p*-value
Amino acids and derivatives	Cr	3.04 (s)	0.9 ± 0.6		1.5 × 10^–3^
	**GSH**	2.96 (m)	3.8 ± 0.9		2.2 × 10^–18^
	**Gln**	2.45 (m)	2.8 ± 0.8		2.1 × 10^–13^
	**Glu**	2.36 (m)	−3.4 ± 0.8		5.6 × 10^–15^
	Gly	3.55 (s)	0.6 ± 0.5		4.9 × 10^–2^
	Ile	0.94 (t)	1.4 ± 0.6		5.5 × 10^–6^
	Lys	1.73 (m)	−1.2 ± 0.6		1.3 × 10^–4^
	Met ^†^	2.64 (t)	2.6 ± 0.7		1.3 × 10^–8^
	**PCr**	3.05 (s)	2.2 ± 0.7		3.1 × 10^–10^
	Phe	7.33 (m)	−0.7 ± 0.6		1.8 × 10^–2^
	Pro	1.98 (m)	−0.9 ± 0.6		1.2 × 10^–2^
	Sarcosine	2.76 (s)	0.9 ± 0.6		1.5 × 10^–3^
	**Tau**	3.43 (t)	2.4 ± 0.7		1.2 × 10^–11^
	Val	1.05 (d)	0.8 ± 0.6		3.8 × 10^–3^
Choline compounds	Cho	3.20 (s)	−1.7 ± 0.6		1.5 × 10^–7^
	GPC	3.23 (s)	−2.3 ± 0.7		2.3 × 10^–6^
Nucleotides and related metabolites	**Adenine**	8.19 (s)	−2.9 ± 0.8		1.5 × 10^–12^
	**Ado**	8.27 (s)	−3.0 ± 0.8		3.0 × 10^–10^
	ADP	8.54 (s)	−0.8 ± 0.6		1.5 × 10^–2^
	AMP	8.61 (s)	−1.7 ± 0.6		2.5 × 10^–8^
	HX	8.20 (s)	−1.9 ± 0.7		7.6 × 10^–9^
	IMP	8.58 (s)	−2.0 ± 0.7		2.3 × 10^–7^
	Ino, Ado	8.35 (s)	−1.3 ± 0.6		8.4 × 10^–5^
	**NAD**^**+**^	8.43 (s)	−3.4 ± 0.8		3.8 × 10^–10^
	Pseudouridine	7.68 (s)	−0.9 ± 0.6		1.5 × 10^–3^
	UDP	8.01 (d)	−1.0 ± 0.6		1.3 × 10^–4^
	UDP-GlcNAc	5.52 (dd)	−1.2 ± 0.6		4.4 × 10^–5^
	**UDP-Glc/GlcA**	7.95 (d)	−3.2 ± 0.8		3.2 × 10^–14^
	UMP	8.11 (s)	−2.1 ± 0.7		2.4 × 10^–9^
	Uracil	5.81 (d)	−0.9 ± 0.6		3.3 × 10^–3^
	Uridine	7.88 (d)	−1.2 ± 0.6		6.8 × 10^–5^
Organic acids	Acetate	1.92 (s)	−1.6 ± 0.6		1.2 × 10^–6^
	Formate	8.46 (s)	−0.6 ± 0.5		4.0 × 10^–2^
	Fumarate	6.52 (s)	−2.0 ± 0.7		3.4 × 10^–9^
	Lactate	4.10 (q)	−1.0 ± 0.6		6.1 × 10^–4^
	PA	0.90 (s)	−1.4 ± 0.6		8.8 × 10^–6^
Other compounds	DMA	2.73 (s)	2.6 ± 0.7		2.5 × 10^–8^
	*Myo*-Inositol	4.06 (t)	−1.3 ± 0.6		8.4 × 10^–4^

All metabolites remain statistically significant (*p* < 0.05) after False Discovery Rate (FDR) correction

3-letter code for amino acids; *Ado* adenosine, *DMA* dimethylamine, *IMP* inosine monophosphate, *Ino* inosine, *PA* pantothenate, *PCr* phosphocreatine, *UDP* uridine diphosphate, *UMP* uridine monophosphate; other abbreviations as defined in the caption of Fig. 2

Metabolites in bold and underlined show more marked differences in levels (*p* ≤ 10^–10^ and |ES|≥ 2.0)

^†^Tentative assignment

**Fig. 4:**
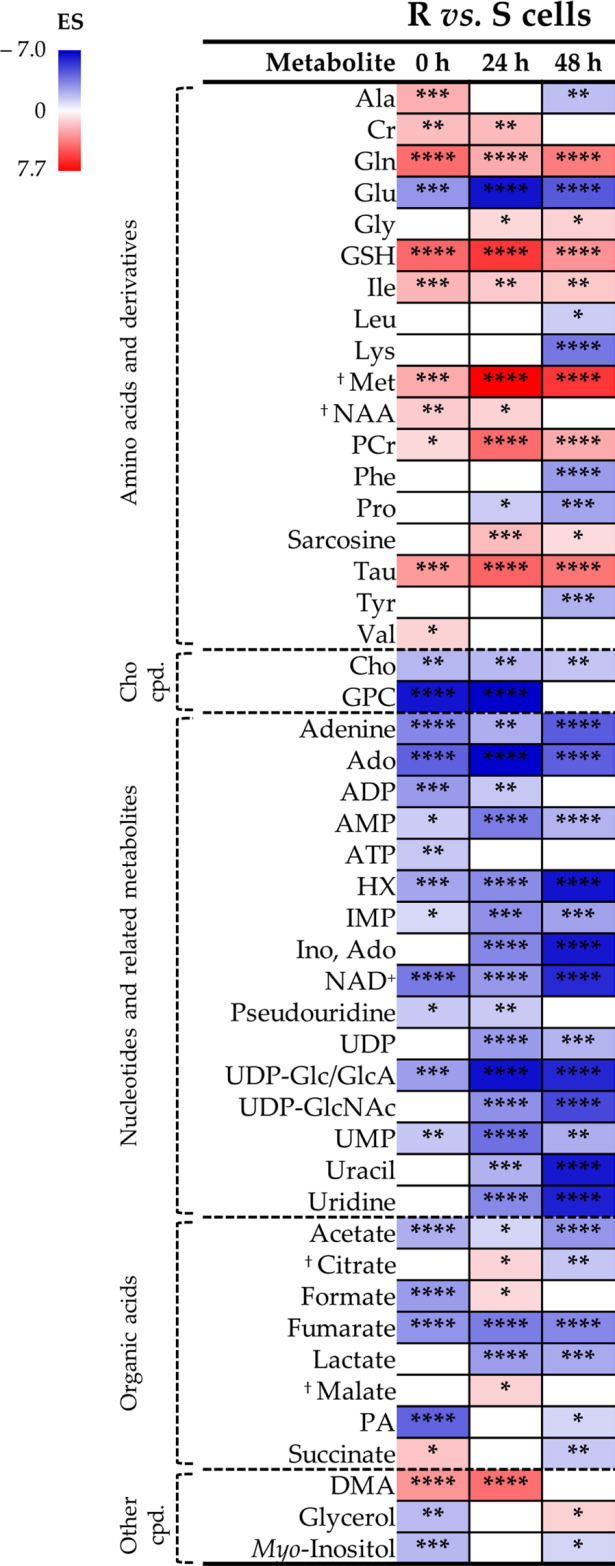
Heatmap significant effect size variations of MDA-MB-231/R (R) compared to MDA-MB-231 (S) considering each time-point. Increasing values of effect size (ES) are colored from blue to red corresponding to negative and positive values, respectively. Abbreviations: Cho, choline; other abbreviations as defined in Fig. 2 and Table 1. ^†^Tentative assignment. *p < 0.05; **p < 0.01; ***p < 0.001; ****p < 0.0001 for the comparison R *vs.* S cells in each time-point

The average differences between R and S cell lines have a perceptible dependence on culture time (Fig. 4), with a general tendency for higher amino acid levels at 0 and 24 h in R cells (except for glutamate and proline), with several amino acids showing depletion at 48 h in the same cells. It is clear that R cells are markedly depleted in choline compounds and in many nucleotides and derivatives, at all time points (Fig. 4). Notably, analysis of distinct time points unveils several new variations in addition to the overall average changes listed in Table 1, namely in (i) tricarboxylic acid cycle (TCA) intermediates citrate, malate and succinate; (ii) amino acids alanine, leucine, *N-*acetyl-aspartate (NAA) and tyrosine; (iii) other compounds: ATP and glycerol. Hence, it is clear that, not only average metabolite pools are significantly different between the two cell lines, but also that time point comparisons unveil additional varying metabolites.

### Metabolic trajectories (0–48 h) in MDA-MB-231/R (R) compared to MDA-MB-231 (S) cells

**Fig. 5:**
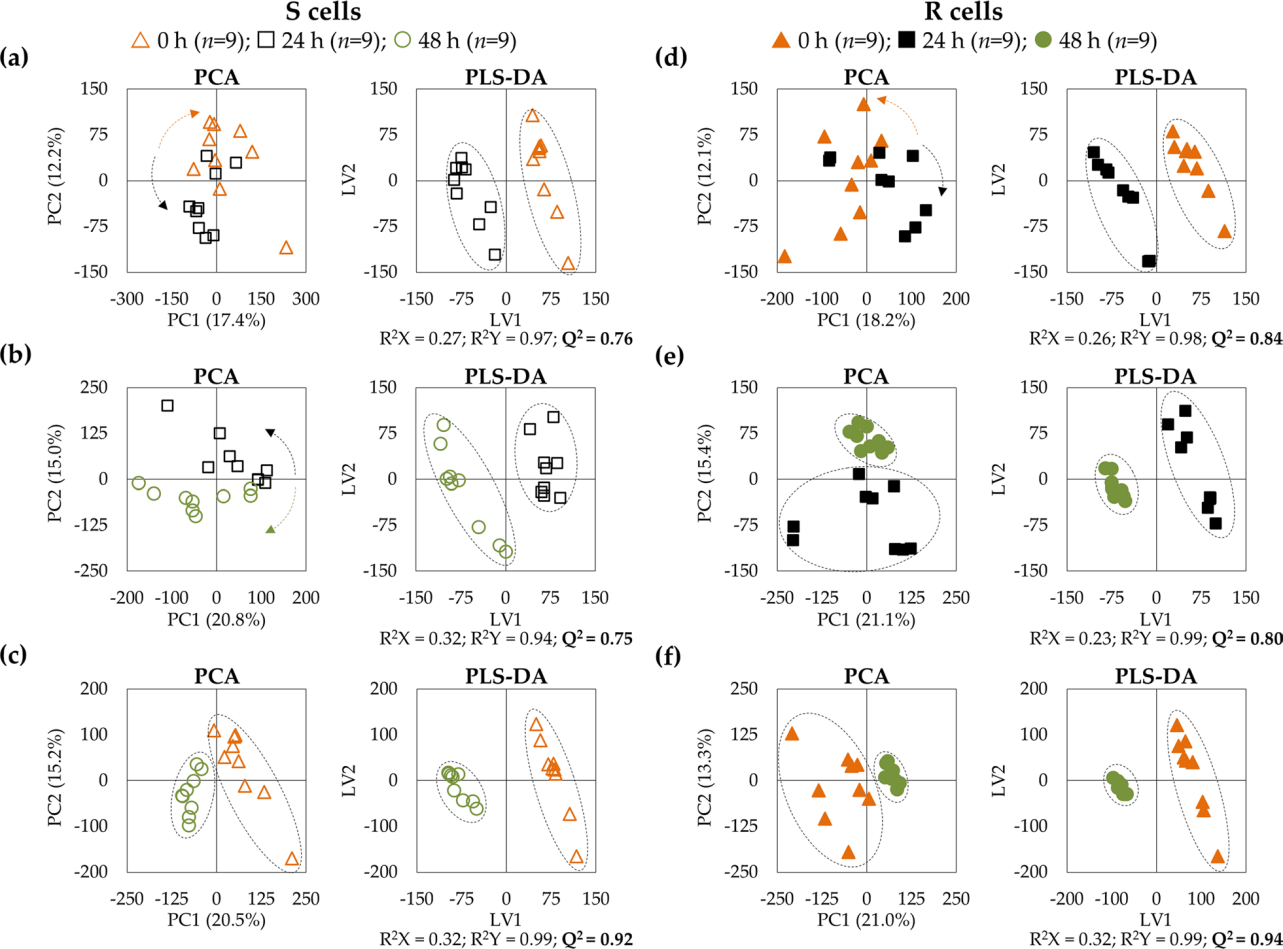
Multivariate analysis of time course variations in MDA-MB-231 cells (S) and MDA-MB-231/R cells (R). PCA and PLS-DA scores scatter plots for ^1^H NMR spectra of aqueous extracts of MDA-MB-231 (S, left) and MDA-MB-231/R (R, right) cells, obtained for time-course pairwise analysis: **a, d** 24 h (black symbols) (black) *vs.* 0 h (orange symbols); **b**, **e** 48 h (green symbols) *vs.* 24 h (black symbols); **c**, **f** 48 h (green symbols) *vs.* 0 h (orange symbols). Validation parameters (R^2^ and Q^2^) are indicated for each PLS-DA model and robust predictive power (Q^2^) are highlighted in bold

In order to examine each metabolite trajectory overtime, pairwise PLS-DA models were obtained for each cell line, revealing high predictive power values (Q^2^) of *ca.* 0.75 and 0.80–0.84 for S and R cells, respectively (Fig. 5a, b) and thus confirming that culture time significantly changes the metabolic profile of each cell line. In particular, this knowledge is relevant for drug exposure studies, where time course markers may be defined. Indeed, at 48 h, the metabolic profile of each cell line is markedly distinct from that at 0 h, with Q^2^ approaching the maximum value of 1.0 (0.92–0.94) (Fig. 5c). The metabolite changes taking place as a function of time (and for the 0/48 h comparison) are quantified in Additional file 2 and Additional file 3 for S and R cell lines, respectively (most changes surviving FDR correction), and illustrated for each cell line in a heatmap form (Additional file 4). In general, both S and R cell lines exhibit a general increase in amino acids from 0 to 24 h (except for glycine which decreases in S cells), followed by a tendency for amino acid stabilization from 24 to 48 h (less number of variations, particularly in R cells). Considering the respective errors (Additional file 2 and Additional file 3), the most evident net differences (48 h *vs* 0 h) in R cells, compared to S cells, comprise: (i) lower/no increases in alanine, isoleucine, leucine and (*N-*acetyl-aspartate) NAA; (ii) more marked increases in creatine (Cr), methionine and taurine. Resistant cells show raised choline levels (no changes in S cells) and no change in GPC (decreased in S cells). The nucleotides profile of R cells is remarkably stable compared to S cells (Additional file 4), only 6 metabolites changing (against 16 in S cells), either in the same direction but lower magnitude then for S cells (ADP, hypoxanthine (HX), UDP and uridine) or in opposite direction *i.e.* decreased in R cells (NAD^+^ and uridine diphosphate *N*-acetyl-glucosamine (UDP-GlcNAc)) (Additional file 4). The S cells exhibit contrastingly strong overtime variations for adenine and uracil and derivatives (Additional file 4). As to organic acids, the R cells show similar trajectories to S cells (decreased acetate and formate, and increased fumarate, lactate and malate), although without changes in citrate, panthothenate (PA) and succinate as observed in S cells (Additional file 4). Glycerol hardly varies in R cells (strongly decreased in S cells), whereas *m-*inositol increases more clearly in R cells) (Additional file 4).

**Fig. 6:**
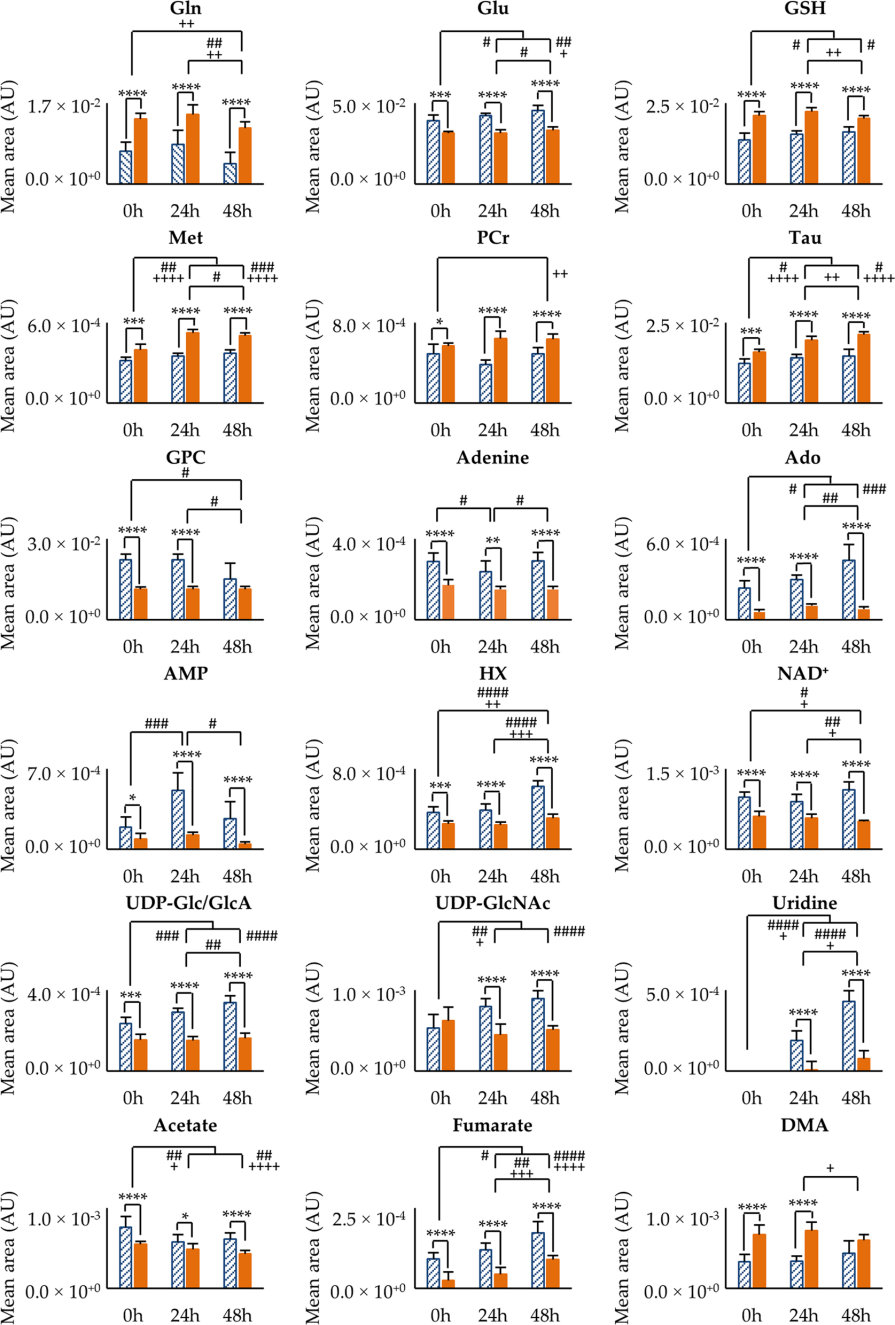
Bar charts of relevant metabolite variations. Significant variations (*p* < 0.001/0.0001 at least in two time-points) on the polar metabolomes of MDA-MB-231 (blue stripes) and MDA-MB-231/R (orange) cells are shown during time-course evolution. Values are expressed as mean of normalized area of integrated peak ± SEM. Abbreviations as defined in Fig. 2 and Table 1. *p < 0.05; **p < 0.01; ***p < 0.001; ****p < 0.0001

**Fig. 7:**
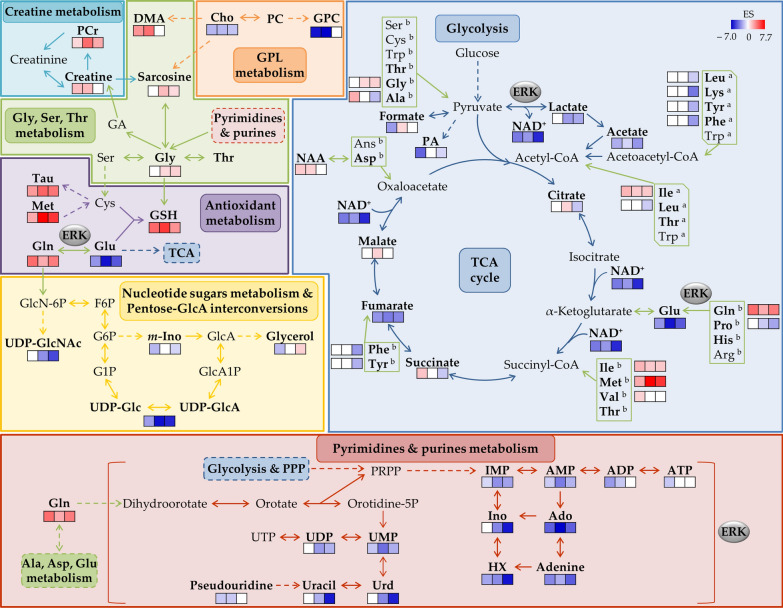
Putative metabolic pathways explaining the differences between polar metabolomes of MDA-MB-231/R and MDA-MB-231 cell lines. Metabolites in bold represent those identified by NMR. Changes in metabolite levels are illustrated as color bars according to the effect size variations for the R vs. S cells pairwise comparison (blue and red represent decreases and increases, respectively). Anaplerotic amino acids classified as ketogenic or glucogenic are indicated with ^a^(within rectangles with cut vertexes) or ^b^(within rectangles with straight vertexes), respectively. Possible relationships with the ERK(/MAPK) signaling pathway are indicated in grey ellipses. Metabolic pathways are distinguished by different colors, namely creatine metabolism in cyan, amino acids metabolism in green, glycerophospholipids (GPL) metabolism in orange, energy metabolism in blue, antioxidant metabolism in purple, nucleotide sugars metabolism & pentose-glcA interconversions in yellow, and pyrimidine & purines metabolism in red. *F6P* fructose 6-phosphate, *G1P* glucose 1-phosphate, *G6P* glucose 6-phosphate, *GA* guanidine acetate, *GlcA1P* glucuronate 1-phosphate, *GlcA* glucuronate, *GlcN-6P* glucosamine 6-phosphate, *PPP* pentose phosphate pathway, *PRPP* phosphoribosyl pyrophosphate, *Urd* uridine, *UTP* uridine

The trajectory graphs (Additional file 5, Additional file 6, Additional file 7) of all varying metabolites simultaneously highlight differences in levels and trajectories. Within these, the strongest distinguishing metabolites (defined as differing at least in two time points with *p* ≤ 0.0001) are shown in Fig. 6. Resistant cells are clearly differentiated at all time points by richer pools of glutamine, GSH, methionine, PCr (leading to higher PCr/Cr ratios at longer times, Additional file 8a), taurine and dimethylamine (DMA); and depleted pools of glutamate, GPC, adenine, adenosine, AMP, HX, NAD^+^, UDP-Glc/GlcA, UDP-GlcNAc, uridine (barely detectable in resistant cells), acetate and fumarate (Fig. 6). Consequently, R cells exhibit consistently lower glutamate/glutamine, GPC/PC, GPC/Cho and NAD^+^/NADH ratios than S cells (Additional file 8). Although ATP and ADP start off depleted in R cells (0 h), both metabolites evolve to comparable levels at 48 h in both cell lines and ADP/ATP significantly distinguishing cell lines only at 24 h (lower ADP/ATP ratio in R cells). The full set of metabolite changes in R cells, compared to S cells, and their dependence with culture time are represented in Fig. 7.

## Discussion

### Cytotoxic, microscopic and biochemical characteristics of MDA-231/R (R) and MDA-MB-231 (S) cells

The cytotoxicity results currently obtained clearly illustrate the high cDDP-resistance of R cells as compared to S cells. This evidence is accompanied by the presence of a higher number of PGCCs in the resistant cells, confirming previous reports of a relationship between the number of PGCCs with tumor chemo-resistance and aggressiveness [45, 46]. PGCCs are cells with multiple nuclei or a single giant nucleus containing multiple complete sets of chromosomes and it is well documented that these cells are present in several solid tumors, usually in lower numbers in sensitive cells before treatment, compared to resistant cells [45]. Moreover, a positive correlation has been formerly described between the number of PGCCs and glioma stage and grade [47].

Furthermore, a basal phosphorylation of NF-κB is observed in S and R cells, although in the R cells a tendency to an increase in p-NF-κB is observed consistent with a slight higher activation of the NF-κB pathway and, hence, cell survival, as shown by reports of the relationship between NF-κB pathway activation, through the IKKα pathway, and cell survival under conditions of cDDP exposure [48]. In addition, a significant decrease in the basal levels of phosphorylated ERK1/2, compared to S cells, seems to characterize the resistance of TNBC cells to cDDP. Again, this is consistent with previous reports associating cancer cells (*e.g.* cervical carcinoma) (in)sensitivity to cDDP with the downregulation of ERK pathway activation [49, 50]. Considering that the ERK1/2 pathway may crosstalk with other signaling pathways, and that reports are found on both ERK 1/2 and the NF-κB being involved in cDDP-acquired resistance in BC cells [51, 52], we propose that the constitutive activation of NF-κB and of ERK1/2 pathways is modified by exposure of S cells to cDDP, with a slightly increase of NF-κB as well as a downregulation of ERK1/2 pathways that might be associated with TNBC survival and its more aggressive characteristics. A similar hypothesis has been advanced in cervical carcinoma, for which increased cDDP-resistance has been observed in association with reduction of activation of the MEK to ERK2 pathway (in the presence of a MEK1-selective inhibitor, 2’-amino-3’-methoxyflavone) and involving, at least in part, an increase of NF-κB activation [50].

### Comparative metabolic features of MDA-MB-231/R (R) and MDA-MB-231 (S) cells

Two of the main metabolic features that R cells maintain overtime is lower anaerobic glycolytic activity (lower lactate levels, although increasing with time in each cell line), and lower glutaminolytic activity (lower glutamate and high glutamine levels). In S cells, both processes are more active, at all times, an expected hallmark of cancer metabolism [53]. However, interestingly, both pathways seem subdued in resistant cells. This seems to contradict the increases in glycolytic and glutaminolytic activities reported in relation to Pt(II)-resistance in colon and ovarian cancer cells [12, 15–17]. We therefore suggest that reduced glycolytic and glutaminolytic activities may be a specific characteristic of TNBC cDDP-sensitive cells. Both observations are consistent with the noted significant decrease in ERK activity. The role of ERK (and JNK) pathways in the metabolic reprogramming of highly proliferating cells (such as cancer cells) has previously been discussed in detail [54]. These authors advance that increased ERK activity accompanies a high proliferative activity in cancer cells leading to a negative regulation of the enzyme pyruvate kinase isoform M2 (PKM2, responsible for PEP to pyruvate conversion), through phosphorylation. This low PKM2 activity induces an accumulation of upstream glycolytic intermediates, which are precursors of several biomolecules (*e.g.* amino acids, nucleotides and fatty acids). Interestingly and paradoxically, a low PKM2 activity is also related with an increased pyruvate conversion into lactate, through the action of lactate dehydrogenase A, at the expense of NADH which is oxidized to NAD^+^. This behavior seems to be descriptive of the cDDP-sensitive cells presently studied, whereas the R cells exhibit all opposite features: decreased ERK activity, decreased lactate formation and NAD^+^ levels (compared to S cells). A similar reasoning applies to the relationship between the ERK signaling pathway and glutaminolysis [54], as a decreased ERK activity in R cells should promote decreased expression of the c-Myc transcription factor, known to lead to lower glutaminolysis activity. Articulation with NF-κB expression (slightly higher in R cells), known to also affect glutaminolysis (as well as glycolysis and OXPHOS [55]), may help to keep glutamine pools high. Glutamine may not only serve glutaminolysis for anaplerosis (which is presently seen to be slowed down in R cells), but is also related to cancer cell stemness [56], a feature which has been generally related to therapy resistance, tumor dormancy and metastatic behavior [57]. As glutamine deprivation has been observed to relate to decreased stemness properties [56], we hypothesize that the richer glutamine pool found here in TNBC cDDP-resistant cells suggests a higher stemness capacity and, hence, a higher cell adaptability (and survival) once under cDDP exposure.

As mentioned before, low glutaminolysis and, thus, low levels of glutamate in R cells should contribute to lower TCA activity, as conversion of glutamate into α-ketoglutarate should decrease. However, it is interesting to note that a decrease in other anaplerotic amino acids, particularly at later culture times, (proline from 24 h; alanine, leucine, phenylalanine, tyrosine and particularly lysine, at 48 h) may suggest their preferential use as precursors into TCA intermediates and pyruvate precursor, respectively (Fig. 7), compared to methionine and branched-chain amino acids (BCAAs) isoleucine and valine, which exhibit higher levels in R cells. BCAA metabolism has been related to cancer resistance in general [58], but it is interesting to note the different leucine behavior in R cells: decreased, as opposed to increased levels of isoleucine and valine. Indeed, leucine metabolism (in particular through the activity of branched-chain amino acid aminotransferase 1, BCAT1) has been recently suggested to lead to activated mTOR-mediated autophagy which, in turn, increases cDDP-resistance [59]. We therefore propose that lower levels of leucine may be related to such mechanism.

Furthermore, the levels of TCA intermediates detected by NMR were strongly dependent on culture time, which suggests a possible modulation of TCA activity overtime. The low NAD^+^ levels (at all time points, and not replenished by lactate production) could either arise from its use in an activated TCA cycle, and/or reflect the general significantly lower availability of nucleotides in R cells, as will be discussed below. Previous reports [54] support the hypothesis that ERK activation promotes aerobic glycolysis and ATP synthesis, subsequently used for phosphorylation. Hence, in R cells, where ERK is less active, ATP synthesis is expected not to be significantly stimulated. Indeed, ATP levels are lower than in S cells at 0 h, although tending towards equivalent levels at later times (note the lower ADP/ATP ratios at 24 h due to ATP increase). We suggest that a later enhanced ATP synthesis in R cells may be a reflection not of ERK activity but, rather, of an adaptive later TCA activation, although the precise dynamics of this pathway overtime requires further investigation, at this stage. These relatively higher ATP levels may also explain the relatively elevated PCr levels (supported by increased Cr and sarcosine, Fig. 7) noted in R cells. Cr to PCr interconversion is an important energy buffer mechanism, which produces high energy PCr particularly in cells with high requirements of energy such as cancer cells [60]. Furthermore, the PCr/Cr ratio has been related to metastasis and proliferation and we advance that a higher PCr/Cr ratios may be related to higher cDDP-resistance.

In addition, increased levels of taurine (the oxidized form of hypotaurine) and GSH are clear discriminators of R cells, indicating an interesting interplay of compounds related to antioxidant protection mechanisms, including methionine (related to taurine through cysteine, and to GSH through the transsulfuration pathway [61] (Fig. 7). Although reactive oxygen species (ROS) were not quantified in this work, the NF-κB signaling pathway is believed to be closely related to oxidative stress [62]. Hence, we propose that the maintenance of high, nearly-constant, GSH levels in R cells, compared to S cells, along with high increasing taurine levels (whereas taurine remains lower and constant in S cells), may indicate that the antioxidative mechanisms in R cells rely preferably on the hypotaurine/taurine pair, rather than on GSH/GSSG. Furthermore, taurine increase has also been reported in ovarian cDDP-resistant cells [12, 13], possibly due to the overexpression of the taurine transporter (TauT) that leads to intracellular taurine accumulation, which in turn is suggested to result in the inhibition of cDDP uptake [13, 63].

The decrease in ERK activity noted in this work may also be related to the marked overall low levels of nucleotides and several of their derivatives, mainly involving adenine, uracil and inosine (including UDP-Glc/GlcA and uridine diphosphate *N*-acetyl-glucosamine UDP-GlcNAc), which make up a nitrogen-base-depleted metabolic profile in R cells, which is more stable overtime than in S cells (Fig. 7). Indeed, a decreased ERK activity and its correlation with increased PKM2 activity may explain the decreased biosynthesis of nucleotides, as described above [54]. However, lower levels of nucleotides may also be related with their use as building blocks as dNTPs to support nucleotide excision repair (NER). This has been reported as a major resistance mechanism against cDDP in several types of cancer [64, 65] and is believed to justify cell death when repairs are not possible [66]. Additionally, poly(ADP-ribose) polymerase (PARP) enzymes, especially PARP1, may determine DNA damage response and maintenance of genome stability through their involvement in NER [as well as in other mechanisms such as base excision repair (BER) and homologous recombination (HR)] [67]. Therefore, we hypothesize that PARP enzymes may have an active role in a more efficient DNA repair in R cells. Upon exposure, these enzymes may interfere with the formation of cDDP-adducts with DNA’s purine bases (adenine and guanine). We hypothesize that the setup of the R cell line, through exposure to low concentrations of cDDP, may have activated these enzymes. As PARP proteins require NAD^+^ to act [67, 68], it is possible that the low NAD^+^ levels in R cells may also reflect this mechanism.

Finally, R cells are also depleted in choline and GPC, the former increasing overtime, while GPC remains stable. This reflects disturbances in membrane metabolism but the exact variation pattern contrasts with results characterizing cDDP-resistance in ovarian cancer cells, which were characterized by increased levels of GPC (although confirming decreased levels of choline as described here) [13]. This relationship between choline compounds and the exact nuances of membrane remodeling mechanisms characterizing cDDP-resistance in TNBC remains unclear, at this stage. We furthermore suggest that the changes observed may reflect distinct lipid metabolic features (eventually detectable by lipid metabolomics) characteristic of this type of resistance in TNBC.

## Conclusion

This work compared the metabolic profile of the MDA-MB-231 parental cDDP-sensitive cell line (time course up to 48 h) with that of a derived cDDP-resistant line, the latter characterized by a more than onefold larger IC_50_ (for cDDP at 48 h), higher number of PGCCs, a slighter higher activation of the NF-κB pathway along with about 50% decrease in the ERK pathway activation. These features were accompanied by a very distinct metabolic signature of resistant cells (polar extracts), which included lower glycolytic and glutaminolytic activities, contrary to observed in other cDDP-resistant cancer cell lines. We propose that such inversion may be a characteristic specific of TNBC (at least as viewed through the MDA-MB-231 cell line), possibly linked to richer glutamine pools supporting increased stemness capacity and, hence, higher survival when under cDDP exposure. The TCA cycle dynamics in resistant cells exhibits some time modulation and an apparent activation at 48 h based on anaplerotic aromatic amino leucine, and particularly lysine. Leucine metabolism differs from those of isoleucine and valine (which accumulate in resistant cells), suggesting a relation of leucine with its know role in mTOR-mediated autophagy, with a reflection in increasing cDDP-resistance. We advance possible markers of TNBC cDDP-resistance such as higher PCr/Cr ratios and a predominance of hypotaurine/taurine–mediated antioxidant protective mechanisms, rather than GSH/GSSG mediated mechanisms. Another clear marker of resistant cells profile is a generalized depletion in nucleotides and derivatives, which may derive from ERK-induced decreased biosynthesis of nucleotides in tandem with their enhanced use in improved DNA repair mechanisms, which thus would decrease cell death. We hypothesize that PARP enzymes may have an active role in this process possibly justifying the consistently low NAD^+^ levels in resistant cells. Other distinguishing features of resistant cells include choline compounds and glycerol, which may indicate the importance of membrane remodeling adaptations in cDDP-resistance.

This work generates several possible hypotheses as to the metabolic adaptations accompanying cDDP-resistance and, although requiring biological demonstration, they pave the way to the use of metabolites as markers of resistance and as guidance to develop therapies to reverse resistance.

### Supplementary Information

**Additional file 1. **List of metabolites and corresponding spin systems visibly identified in 500 MHz ^1^H NMR spectra of aqueous extracts of TNBC cells MDA-MB-231 (S) and MDA-MB-231/R (R), sensitive and resistant to cDDP, respectively.

**Additional file 2. **Statistically significant metabolite variations observed in the polar metabolome of MDA-MB-231 during time-course evolution and the two extreme time-points.

**Additional file 3. **Statistically significant metabolite variations observed in the polar metabolome of MDA-MB-231/R during time-course evolution and the two extreme time-points.

**Additional file 4. **Heatmap of the effect size (ES) values of statistically significant variations during the experimental time-courses in both MDA-MB-231 and MDA-MB-231/R cells.

**Additional file 5. **Bar charts illustrating time-course variations for amino acids altered significantly in MDA-MB-231 and MDA-MB-231/R cells.

**Additional file 6. **Bar charts illustrating time-course variations for (a) choline compounds (b) organic acids and (c) other compounds altered significantly in MDA-MB-231 and MDA-MB-231/R cells.

**Additional file 7. **Bar charts illustrating time-course variations for nucleotides and derivatives altered significantly in MDA-MB-231 and MDA-MB-231/R cells.

**Additional file 8. **Bar charts illustrating time-course variations for ratios of (a) Phosphocreatine/Creatine, (b) Glutamate/Glutamine, (c) GPC/PC, (d) GPC/Cho (e) NAD^+^/NADH, and (f) ADP/APT calculated for MDA-MB-231 and MDA-MB-231/R cells.

## Data Availability

The datasets generated and/or analyzed during the current study are available in the Metabolomics Workbench: An international repository for metabolomics data and metadata, metabolite standards, protocols, tutorials and training, and analysis tools (2016), using the website https://www.metabolomicsworkbench.org, and the study ID ST002720.

## Abbreviations

Ado: Adenosine
ADP: Adenosine diphosphate
AMP: Adenosine monophosphate
ATP: Adenosine triphosphate
BC: Breast cancer
BCAA: Branched-chain amino acids
BCAT1: Branched-chain amino acid aminotransferase 1
BER: Base excision repair
Cpd.: Compounds
cDDP: Cisplatin
Cho: Choline
Cr: Creatine
DMA: Dimethylamine
ES: Effect size
F6P: Fructose 6-phosphate
FDR: False discovery rate
G1P: Glucose 1-phosphate
G6P: Glucose 6-phosphate
GA: Guanidine acetate
GlcA1P: Glucuronate 1-phosphate
GlcA: Glucuronate
GlcN-6P: Glucosamine 6-phosphate
GPC: Glycerophosphocholine
GSH: Glutathione (reduced)
GTP: Guanosine triphosphate
HR: Homologous recombination
HX: Hypoxanthine
IMP: Inosine monophosphate
Ino: Inosine
MS: Mass spectrometry
KEGG: Kyoto encyclopedia of genes and genomes
NAA: *N*-Acetyl-aspartate
NAD^+^/NADH: Nicotinamide adenine dinucleotide (oxidized/ reduced)
NER: Nucleotide excision repair
NMR: Nuclear magnetic resonance
OXPHOS: Oxidative phosphorylation
PA: Pantothenate
PARP: Poly(ADP-ribose) polymerase
PC: Phosphocholine
PCA: Principal component analysis
PCr: Phosphocreatine
PGCC: Polyploid giant cancer cell
PKM2: Pyruvate kinase isoform M2
PLS-DA: Partial least squares–discriminant analysis
PPP: Pentose phosphate pathway
PRPP: Phosphoribosyl pyrophosphate
Pt(II): Platinum(II)
ROS: Reactive oxygen species
Tau: Taurine
TauT: Taurine transporter
TCA: Tricarboxylic acid cycle
TMAO: Trimethylamine *N*-oxide
TNBC: Triple-negative breast cancer
TSP: 3-(Trimethylsilyl)-propionic-2,2,3,3-d4 acid
UV: Unit variance
UDP: Uridine diphosphate
UDP-Glc/GlcA: Uridine diphosphate glucose/glucuronate
UDP-GlcNAc: Uridine diphosphate *N*-acetyl-glucosamine
UMP: Uridine monophosphate
Urd: Uridine
UTP: Uridine triphosphate
VIP: Variable importance to the projection
s: Singlet
d: Doublet
dd: Double doublet
t: Triplet
q: Quartet
m: Multiplet
