# Supplementary material for: Disclosing a metabolic signature of cisplatin resistance in MDA-MB-231 triple-negative breast cancer cells by NMR metabolomics
Source: Cancer Cell Int. 2023 Dec 6;23:310. doi: 10.1186/s12935-023-03124-0 (PMC10699005; doi:10.1186/s12935-023-03124-0)
Supplement: Supplementary file 1 — Additional file 1. List of metabolites and corresponding spin systems visibly identified in 500 MHz 1H NMR spectra of aqueous extracts of TNBC cells MDA-MB-231 (S) and MDA-MB-231/R (R), sensitive and resistant to cDDP, respectively. [file 12935_2023_3124_MOESM1_ESM.docx]

**Additional file 1**. List of metabolites and corresponding spin systems visibly identified in 500 MHz ^1^H NMR spectra of aqueous extracts of TNBC cells MDA-MB-231 (S) and MDA-MB-231/R (R), sensitive and resistant to cDDP, respectively. Arrows represent average significant variations in R compared to S cell lines: 🠅, increased levels; 🠇, decreased levels. ^†^ Tentative assignment. Abbreviations: Ado, adenosine; ADP, adenosine diphosphate; AMP, adenosine monophosphate; ATP, adenosine triphosphate; Cpd., compounds; Cho, choline; Cr, creatine; DMA, dimethylamine; GPC, glycerophosphocholine; GSH, glutathione (reduced); GTP, guanosine triphosphate; HX, hypoxanthine; IMP, inosine monophosphate; Ino, inosine; NAA, *N*-acetylaspartate; NAD^+^/NADH, nicotinamide adenine dinucleotide (oxidized/ reduced); PA, pantothenate; PC, phosphocholine; PCr, phosphocreatine; Tau, taurine; TMAO, trimethylamine-*N*-oxide; UDP, uridine diphosphate; UDP-Glc/ GlcA, uridine diphosphate glucose/ glucuronate; UDP-GlcNAc, uridine diphosphate *N*-acetylglucosamine; UMP, uridine monophosphate. Multiplicity abbreviations: s, singlet; d, doublet; dd, double doublet; t, triplet; q, quartet; m, multiplet.

| **Metabolite** | **δ_H_ ppm (multiplicity, assignment)** | **HMDB ID [34]** | **R *vs.* S** |
| --- | --- | --- | --- |
| **Amino acids** |  |  |  |
| Alanine | 1.48 (d, βCH_3_); 3.78 (q, αCH) | HMDB0000161 | - |
| Aspartate | 2.68 (dd, βCH); 2.82 (dd, β’CH); 3.90 (dd, αCH) | HMDB0000191 | - |
| Cr | 3.04 (s, N-CH_3_); 3.93 (s, N-CH_2_) | HMDB0000064 | 🠅 |
| Glutamate | 2.04 (m, βCH); 2.11 (m, β’CH); 2.36 (m, γCH_2_); 3.76 (dd, αCH) | HMDB0000148 | 🠇 |
| Glutamine | 2.14 (m, βCH_2_); 2.45 (m, γCH_2_); 3.77 (t, αCH) | HMDB0000641 | 🠅 |
| Glycine | 3.55 (s, αCH_2_) | HMDB0000123 | 🠅 |
| GSH | 2.17 (m, βCH_2_ Glu); 2.55 (m, γCH_2_ Glu); 2.96 (m, αCH_2_ Cys); 3.78 (αCH Glu); 4.57 (m, βCH_2_ Cys) | HMDB0000125 | 🠅 |
| Histidine | 7.10 (s, C4H ring); 7.98 (s, C2H ring) | HMDB0000177 | - |
| Isoleucine | 0.94 (t, δCH_3_); 1.01 (d, β'CH_3_); 1.99 (m, βCH) | HMDB0000177 | 🠅 |
| Leucine | 0.96 (t, δCH_3_/ δ’CH_3_); 1.71 (m, βCH_2_/ γCH) | HMDB0000687 | - |
| Lysine | 1.45 (m, γCH_2_); 1.73 (m, δCH_2_); 1.92 (m, βCH_2_); 3.02 (t,εCH_2_) | HMDB0000182 | 🠇 |
| Methionine ^†^ | 2.15 (m, βCH_2_); 2.64 (t, γCH_2_) | HMDB0000696 | 🠅 |
| NAA ^†^ | 2.02 (s, CH_3_); 2.68 (m, β’CH_2_) | HMDB0000812 | - |
| Phenylalanine | 7.33 (d, C2H/ C6H ring); 7.38 (m, C4H ring); 7.42 (t, C3H/ C5H ring) | HMDB0000159 | 🠇 |
| PCr | 3.05 (s, N-CH_3_); 3.95 (s, N-CH_2_) | HMDB0001511 | 🠅 |
| Proline | 1.98 (m, γC2H); 3.34 (m, β’C2H) | HMDB0000162 | 🠇 |
| Sarcosine | 2.76 (s, CH_3_); 3.62 (s, CH_2_) | HMDB0000271 | 🠅 |
| Tau | 3.27 (t, S-CH_2_); 3.43 (t, N-CH_2_) | HMDB0000251 | 🠅 |
| Threonine | 1.33 (d, γCH_3_); 3.59 (d, αCH); 4.24 (m, βCH) | HMDB0000167 | - |
| Tyrosine | 6.90 (d, C3H/ C5H ring); 7.20 (d, C2H/ C6H ring) | HMDB0000158 | - |
| Valine | 0.99 (d, γCH_3_); 1.05 (d, γ'CH_3_); 3.61 (d, αCH) | HMDB0000883 | 🠅 |
| **Choline cpd.** | | | |
| Cho | 3.21 (s, N(CH_3_)_3_) | HMDB0000097 | 🠇 |
| GPC | 3.23 (s, N(CH_3_)_3_); 4.33 (m, PO_3_-αCH_2_) | HMDB0000086 | 🠇 |
| PC | 3.22 (s, N(CH_3_)_3_); 3.60 (t, N-CH_2_); 4.17 (m, PO_3_-CH_2_) | HMDB0001565 | - |
| **Nucleotides and derivatives** | | | |
| Adenine | 8.19 (s, C8H ribose); 8.23 (s, C2H ring) | HMDB0000034 | 🠇 |
| Ado | 4.29 (q, C4’H ribose); 4.44 (dd, C3’H ribose); 6.10 (d, C1’H ribose); 8.27 (s, C2H ring) | HMDB0000050 | 🠇 |
| ADP | 6.15 (d, C1’H ribose); 8.27 (s, C2H ring); 8.54 (s, C8H ring) | HMDB0001341 | 🠇 |
| AMP | 6.14 (d, C1’H ribose); 8.27 (s, C2H ring); 8.61 (s, C8H ring) | HMDB0000045 | 🠇 |
| ATP | 6.15 (d, C1’H ribose); 8.28 (s, C2H ring); 8.55 (s C8H ring) | HMDB0000538 | - |
| GTP ^†^ | 5.94 (d, C1’H ribose); | HMDB0001273 | - |
| HX | 8.18 (s, C2H); 8.20 (s, C8H) | HMDB0000157 | 🠇 |
| IMP | 8.24 (s, C8H ring); 8.58 (s, C8H ring) | HMDB0000175 | 🠇 |
| Ino | 6.10 (d, C1’H); 8.24 (s, C8H ring); 8.35 (s, C2H ring) | HMDB0000195 | 🠇 |
| NAD^+^ | 6.04 (d, N1’); 8.18 (s, A2); 8.19 (N5); 8.43 (s, A8); 8.83 (d, N4); 9.15 (d, N6); 9.34 (s, N2) | HMDB0000902 | 🠇 |
| NADH | 5.98 (dd, N6); 8.25 (s, A2), 8.48 (s, A8);8.48 (s, A8) | HMDB0001487 | - |
| Pseudouridine | 7.68 (s, C6H) | HMDB0000767 | 🠇 |
| UDP | 5.97 (m, C6H ring); 8.01 (d, C5H ring) | HMDB0000295 | 🠇 |
| UDP-Glc/ GlcA | 5.61 (dd, C1H glucose); 7.95 (d, C2H uridine) | HMDB0000935 | 🠇 |
| UDP-GlcNAc | 5.52 (dd, C1H glucose); 5.95 (d, C5H uridine) | HMDB0000290 | 🠇 |
| UMP | 5.99 (m, C6H ring); 8.11 (d, C5H ring) | HMDB0000288 | 🠇 |
| Uracil | 5.81 (d, C6H); 7.54 (d, C5H) | HMDB0000300 | 🠇 |
| Uridine | 5.90 (d, C5H ring); 5.92 (d, C1’H ring); 7.88 (d, C6H ring) | HMDB0000285 | 🠇 |
| **Organic acids** |  |  |  |
| Acetate | 1.92 (s, CH_3_) | HMDB0000042 | 🠇 |
| Citrate ^†^ | 2.70 (d, α’/β’CH2) | HMDB0000094 | - |
| Formate | 8.46 (s, CH) | HMDB0000142 | 🠇 |
| Fumarate | 6.52 (s, CH) | HMDB0000134 | 🠇 |
| Lactate | 1.33 (d, CH_3_); 4.10 (q, CH) | HMDB0000190 | - |
| Malate ^†^ | 2.67 (dd, βCH); 4.30 (dd, αCH) | HMDB0000156 | - |
| PA | 0.90 (s, CH_3_); 0.94 (s, CH_3_) | HMDB0000210 | 🠇 |
| Succinate | 2.41 (s, CH_2_) | HMDB0000254 | - |
| **Other cpd.** |  |  |  |
| DMA | 2.73 (s, (CH_3_)_2_) | HMDB0000087 | 🠅 |
| Glycerol | 3.56 (dd, C1H_2_/C3H_2_); 3.65 (dd, C1’H_2_/C3’H_2_); 3.78 (m, C2H) | HMDB0000131 | - |
| *m*-Inositol | 3.28 (t, C5H); 3.53 (dd, C1H/ C3H); 3.62 (t, C4H/ C6H); 4.06 (t, C2H) | HMDB0000211 | 🠇 |
| TMAO | 3.27 (s, CH_3_) | HMDB0000925 | - |
