# Supplementary material for: Disclosing a metabolic signature of cisplatin resistance in MDA-MB-231 triple-negative breast cancer cells by NMR metabolomics
Source: Cancer Cell Int. 2023 Dec 6;23:310. doi: 10.1186/s12935-023-03124-0 (PMC10699005; doi:10.1186/s12935-023-03124-0)
Supplement: Supplementary file 2 — Additional file 2. Statistically significant metabolite variations observed in the polar metabolome of MDA-MB-231 during time-course evolution and the two extreme time-points. [file 12935_2023_3124_MOESM2_ESM.docx]

**Additional file 2.** Statistically significant (|ES| > ES Error and *p*-value < 0.05) metabolite variations observed in the polar metabolome of MDA-MB-231 during time-course evolution (0 h *vs.* 24 h and 24 h *vs.* 48 h, left and middle columns, respectively) and the two extreme time-points (0 h *vs.* 48 h, right column). ^†^ Tentative assignment. ^a^ Metabolic variation not statistically significant after False Discovery Rate (FDR) correction (*p*-value > 0.05). Abbreviations: 3-letter code used for amino acids; other abbreviations as defined in Additional file 1.

|  | **Metabolite** | **δ_H_ (multiplicity)** | **24 h *vs.* 0 h** | | | **48 h *vs.* 24 h** | | | **48 h *vs.* 0 h** | | |
| --- | --- | --- | --- | --- | --- | --- | --- | --- | --- | --- | --- |
|  |  |  | ES ± | Error | *p*-value | ES ± | Error | *p*-value | ES ± | Error | *p*-value |
| Amino acids and derivatives &  choline compounds | Ala | 1.48 (d) | 2.3 ± | 1.2 | 4.9 × 10^-4^ | — |  | — | 4.4 ± | 1.8 | 2.0 × 10^-7^ |
|  | Asp | 2.82 (dd) | 1.3 ± | 1.0 | 1.5 × 10^-2^ | — |  | — | — |  | — |
|  | Cr | 3.04 (s) | 1.3 ± | 1.0 | 1.2 × 10^-2^ | 1.2 ± | 1.0 | 2.3 × 10^-2^ | 2.2 ± | 1.2 | 3.5 × 10^-4^ |
|  | Gln | 2.45 (m) | — |  | — | – 1.5 ± | 1.0 | 3.9 × 10^-3^ | — |  | — |
|  | Glu | 2.36 (m) | 1.3 ± | 1.0 | 2.1 × 10^-2^ | 1.2 ± | 1.0 | 2.9 × 10^-2^ | 1.9 ± | 1.1 | 1.1 × 10^-3^ |
|  | Gly | 3.55 (s) | – 1.2 ± | 1.0 | 2.6 × 10^-2^ | — |  | — | – 1.4 ± | 1.0 | 9.9 × 10^-3^ |
|  | GSH | 2.96 (m) | 1.1 ± | 0.9 | 4.6 × 10^-2^ **^a^** | — |  | — | 1.3 ± | 1.0 | 1.3 × 10^-2^ |
|  | Ile | 0.94 (t) | 1.4 ± | 1.0 | 1.4 × 10^-2^ | — |  | — | 2.1 ± | 1.1 | 5.1 × 10^-4^ |
|  | Leu | 0.96 (t) | 1.1 ± | 1.0 | 2.4 × 10^-2^ | — |  | — | 2.4 ± | 1.2 | 4.1 × 10^-5^ |
|  | Lys | 1.73 (m) | — |  | — | 2.0 ± | 1.1 | 6.5 × 10^-4^ | 2.1 ± | 1.1 | 6.2 × 10^-4^ |
|  | Met ^†^ | 2.64 (t) | 1.4 ± | 1.0 | 8.8 × 10^-3^ | 1.1 ± | 1.0 | 3.3 × 10^-2^ | 2.3 ± | 1.2 | 1.5 × 10^-4^ |
|  | NAA ^†^ | 2.02 (s) | 1.0 ± | 0.9 | 4.8 × 10^-2^ **^a^** | 3.9 ± | 1.6 | 5.5 × 10^-7^ | 4.1 ± | 1.6 | 1.3 × 10^-6^ |
|  | Phe | 7.33 (m) | 2.3 ± | 1.2 | 1.6 × 10^-4^ | 1.0 ± | 0.9 | 4.0 × 10^-2^ **^a^** | 3.6 ± | 1.5 | 4.1 × 10^-5^ |
|  | Pro | 1.98 (m) | 1.2 ± | 1.0 | 2.6 × 10^-2^ | 2.0 ± | 1.1 | 1.4 × 10^-3^ | 2.6 ± | 1.3 | 1.6 × 10^-4^ |
|  | Tau | 3.43 (t) | 1.3 ± | 1.0 | 1.3 × 10^-2^ | — |  | — | 1.2 ± | 1.0 | 1.9 × 10^-2^ |
|  | Tyr | 7.20 (d) | 1.9 ± | 1.1 | 1.4 × 10^-3^ | — |  | — | 3.5 ± | 1.5 | 1.9 × 10^-6^ |
|  | Val | 1.05 (d) | 1.2 ± | 1.0 | 2.4 × 10^-2^ | — |  | — | 2.1 ± | 1.1 | 6.0 × 10^-4^ |
|  | GPC | 3.23 (s) | — |  | — | – 1.6 ± | 1.1 | 3.2 × 10^-2^ | – 1.6 ± | 1.1 | 2.4 × 10^-2^ |
| Nucleotides and derivatives | Adenine | 8.19 (s) | – 1.0 ± | 0.9 | 4.4 × 10^-2^ **^a^** | 1.1 ± | 1.0 | 2.9 × 10^-2^ | — |  | — |
|  | Ado | 8.27 (s) | 1.3 ± | 1.0 | 1.6 × 10^-2^ | 1.7 ± | 1.1 | 5.2 × 10^-3^ | 2.3 ± | 1.2 | 4.9 × 10^-4^ |
|  | ADP | 8.54 (s) | -3.6 ± | 1.5 | 4.1 × 10^-5^ | -1.9 ± | 1.1 | 1.2 × 10^-3^ | – 6.0 ± | 2.2 | 4.6 × 10^-9^ |
|  | AMP | 8.61 (s) | 2.6 ± | 1.2 | 1.2 × 10^-4^ | -1.6 ± | 1.1 | 1.1 × 10^-2^ | — |  | — |
|  | ATP | 8.55 (s) | – 2.5 ± | 1.2 | 1.7 × 10^-4^ | — |  | — | – 6.3 ± | 2.2 | 5.1 × 10^-10^ |
|  | HX | 8.20 (s) | — |  | — | 4.0 ± | 1.6 | 2.9 × 10^-7^ | 4.4 ± | 1.7 | 6.6 × 10^-8^ |
|  | IMP | 8.58 (s) | 1.3 ± | 1.0 | 1.5 × 10^-2^ | — |  | — | — |  | — |
|  | Ino, Ado | 8.35 (s) | 3.5 ± | 1.5 | 1.7 × 10^-6^ | 4.1 ± | 1.6 | 6.7 × 10^-7^ | 6.8 ± | 2.4 | 1.8 × 10^-9^ |
|  | NAD^+^ | 8.43 (s) | — |  | — | 1.6 ± | 1.1 | 3.6 × 10^-3^ | 1.2 ± | 1.0 | 2.3 × 10^-2^ |
|  | Pseudouridine | 7.68 (s) | — |  | — | – 1.2 ± | 1.0 | 2.1 × 10^-2^ | – 1.4 ± | 1.0 | 1.6 × 10^-2^ |
|  | UDP | 8.01 (d) | — |  | — | – 2.2 ± | 1.2 | 3.5 × 10^-4^ | – 1.9 ± | 1.1 | 1.2 × 10^-3^ |
|  | UDP-GlcNAc | 5.52 (dd) | 1.9 ± | 1.1 | 1.3 × 10^-3^ | — |  | — | 2.7 ± | 1.3 | 8.8 × 10^-5^ |
|  | UDP-Glc/GlcA | 7.95 (d) | 2.1 ± | 1.2 | 5.8 × 10^-4^ | 1.7 ± | 1.1 | 2.8 × 10^-3^ | 3.2 ± | 1.4 | 4.3 × 10^-6^ |
|  | UMP | 8.11 (s) | 1.4 ± | 1.0 | 1.4 × 10^-2^ | — |  | — | — |  | — |
|  | Uracil | 5.81 (d) | 2.4 ± | 1.2 | 1.7 × 10^-4^ | 3.4 ± | 1.4 | 4.1 × 10^-5^ | 5.0 ± | 1.9 | 4.1 × 10^-5^ |
|  | Uridine | 7.88 (d) | 2.8 ± | 1.3 | 3.9 × 10^-5^ | 3.9 ± | 1.6 | 4.3 × 10^-7^ | 5.8 ± | 2.1 | 4.2 × 10^-9^ |
| Organic acids | Acetate | 1.92 (s) | – 1.6 ± | 1.1 | 2.8 × 10^-3^ | — |  | — | – 1.3 ± | 1.0 | 5.6 × 10^-3^ |
|  | Citrate ^†^ | 2.70 (d) | — |  | — | 1.9 ± | 1.1 | 1.9 × 10^-3^ | 1.9 ± | 1.1 | 1.6 × 10^-3^ |
|  | Formate | 8.46 (s) | – 3.8 ± | 1.5 | 1.6 × 10^-6^ | — |  | — | – 2.8 ± | 1.3 | 2.1 × 10^-5^ |
|  | Fumarate | 6.52 (s) | 1.4 ± | 1.0 | 1.1 × 10^-2^ | 1.9 ± | 1.1 | 1.3 × 10^-3^ | 2.9 ± | 1.3 | 3.1 × 10^-5^ |
|  | Lactate | 4.10 (q) | 1.8 ± | 1.1 | 2.7 × 10^-3^ | 2.2 ± | 1.2 | 3.3 × 10^-4^ | 3.2 ± | 1.4 | 3.0 × 10^-5^ |
|  | Malate ^†^ | 2.67 (dd) | — |  | — | 2.3 ± | 1.2 | 2.1 × 10^-4^ | 2.7 ± | 1.3 | 2.9 × 10^-5^ |
|  | PA | 0.90 (s) | — |  | — | — |  | — | – 1.6 ± | 1.1 | 5.4 × 10^-3^ |
|  | Succinate | 2.41 (s) | 1.8 ± | 1.1 | 2.2 × 10^-3^ | 1.7 ± | 1.1 | 1.7 × 10^-4^ | 2.6 ± | 1.2 | 4.1 × 10^-5^ |
| Other cpd. | Glycerol | 3.65 (dd) | – 2.7 ± | 1.3 | 8.2 × 10^-5^ | – 1.3 ± | 1.0 | 7.8 × 10^-4^ | – 3.5 ± | 1.5 | 6.5 × 10^-5^ |
|  | *Myo*-Inositol | 4.06 (t) | — |  | — | — |  | — | 1.2 ± | 1.0 | 3.2 × 10^-2^ |
