# Supplementary material for: Disclosing a metabolic signature of cisplatin resistance in MDA-MB-231 triple-negative breast cancer cells by NMR metabolomics
Source: Cancer Cell Int. 2023 Dec 6;23:310. doi: 10.1186/s12935-023-03124-0 (PMC10699005; doi:10.1186/s12935-023-03124-0)
Supplement: Supplementary file 4 — Additional file 4. Heatmap of the effect size (ES) values of statistically significant variations during the experimental time-courses in both MDA-MB-231 and MDA-MB-231/R cells. [file 12935_2023_3124_MOESM4_ESM.docx]

**Additional file 4**

**
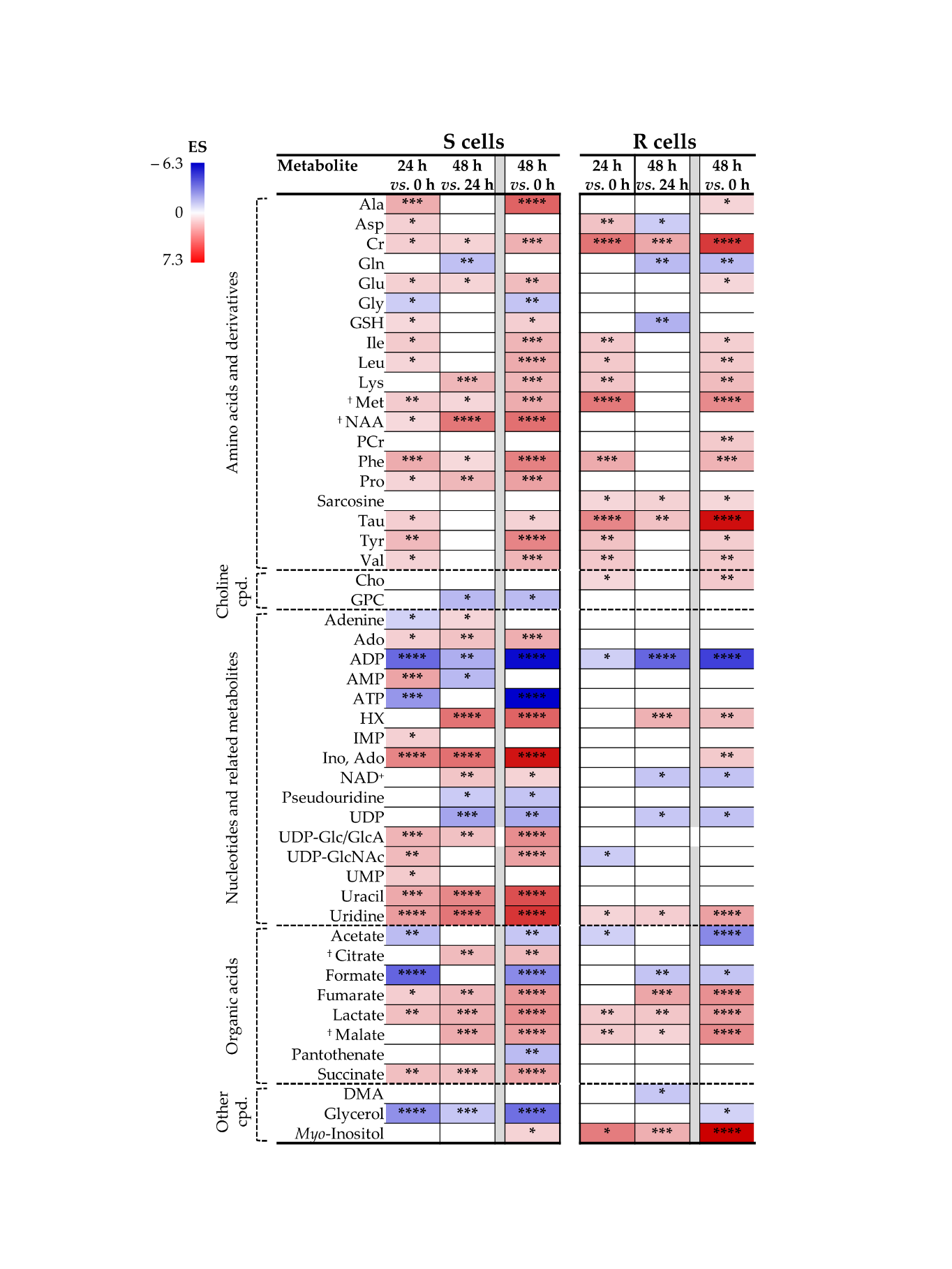
**

**Additional file 4.** Heatmap of the effect size (ES) values of statistically significant variations during the experimental time-courses in both MDA-MB-231 (S) and MDA-MB-231/R (R) cells. Metabolic variations are colored from blue to red representing an increasing ES scale from negative to positive values, respectively. Abbreviations: cdp., compounds; NAA, *N*-acetylaspartate; other abbreviations as defined in Additional file 1. † Tentative assignment. * p-value < 0.05; ** p-value < 0.01; *** p-value < 0.001; **** p-value < 0.0001 for the comparison R vs. S cells in each time-point.
