# Supplementary material for: Disclosing a metabolic signature of cisplatin resistance in MDA-MB-231 triple-negative breast cancer cells by NMR metabolomics
Source: Cancer Cell Int. 2023 Dec 6;23:310. doi: 10.1186/s12935-023-03124-0 (PMC10699005; doi:10.1186/s12935-023-03124-0)
Supplement: Supplementary file 8 — Additional file 8. Bar charts illustrating time-course variations for ratios of (a) Phosphocreatine/Creatine, (b) Glutamate/Glutamine, (c) GPC/PC, (d) GPC/Cho (e) NAD+/NADH, and (f) ADP/APT calculated for MDA-MB-231 and MDA-MB-231/R cells. [file 12935_2023_3124_MOESM8_ESM.docx]

**Additional file 8**

**
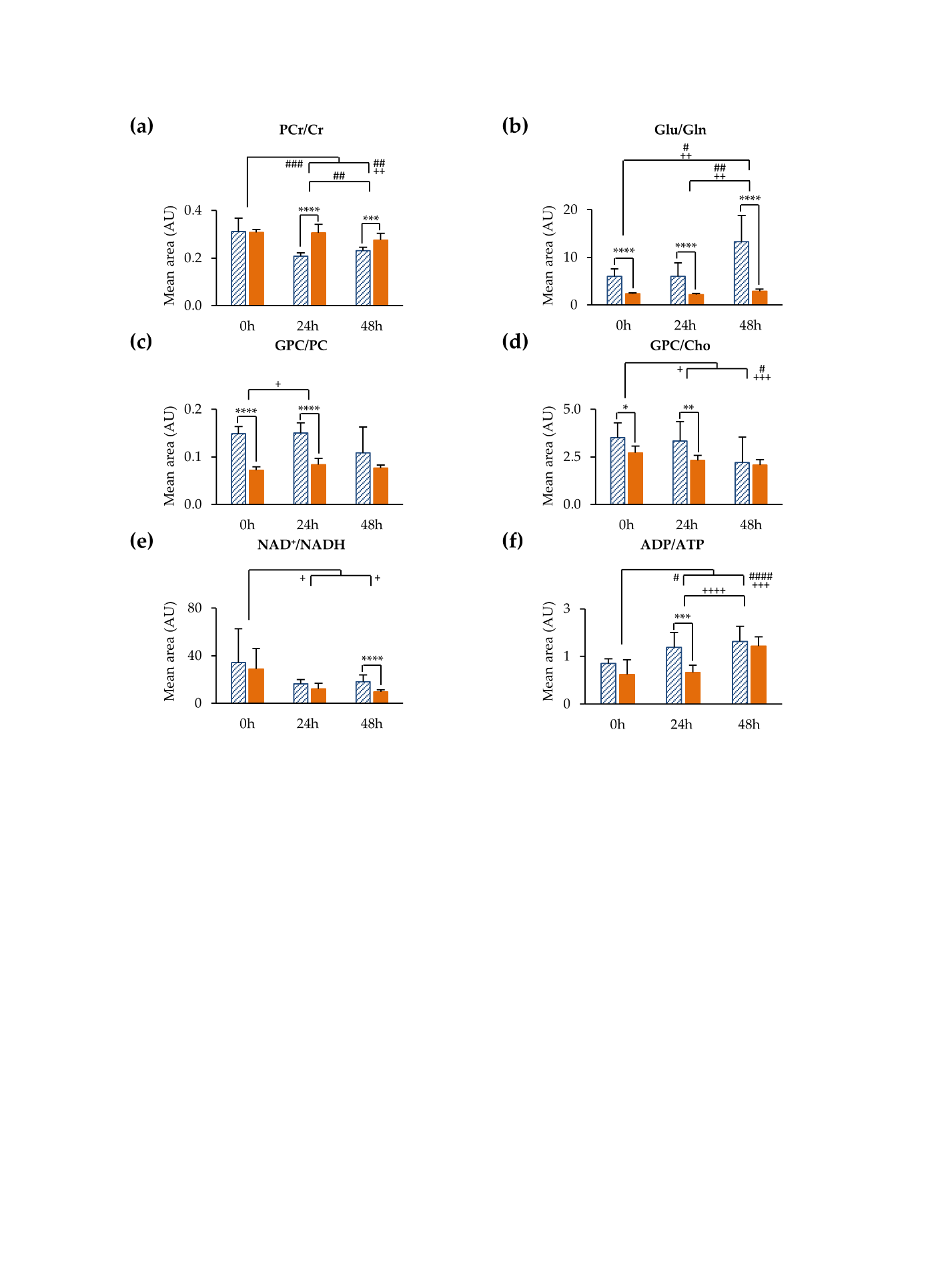
**

**Additional file 8.** Bar charts illustrating time-course variations for racios of **(a)** Phosphocreatine/Creatine, **(b)** Glutamate/Glutamine, **(c)** GPC/PC, **(d)** GPC/Cho **(e)** NAD^+^/NADH, and **(f)** ADP/APT calculated for MDA-MB-231 (blue stripes) *vs.* MDA-MB-231/R (orange) cells. Values are expressed as racios mean of normalized area of integrated peak ± SEM. Abbreviations as defined in Additional file 1. Significant differences in S (+) and R (#) cells trajectory along 0, 24 and 48 h, respectively: +/# *p*-value < 0.05; ++/## *p*-value < 0.01; +++/### *p*-value < 0.001; ++++/#### *p*-value < 0.0001. Significant differences between R *vs.* S (*) in each time-point: * *p*-value < 0.05; ** *p*-value < 0.01; *** *p*-value < 0.001; **** *p*-value < 0.0001.
